# Rh(I)-catalyzed intramolecular [2 + 2 + 1] cycloaddition of allenenes: Construction of bicyclo[4.3.0]nonenones with an angular methyl group and tricyclo[6.4.0.0^1,5^]dodecenone

**DOI:** 10.3762/bjoc.7.52

**Published:** 2011-04-07

**Authors:** Fuyuhiko Inagaki, Naoya Itoh, Yujiro Hayashi, Yumi Matsui, Chisato Mukai

**Affiliations:** 1 Division of Pharmaceutical Sciences, Graduate School of Natural Science and Technology, Kanazawa University, Kakuma-machi, Kanazawa 920-1192, Japan

**Keywords:** allene, bicyclo[4.3.0] derivatives, carbonylative [2 + 2 + 1] cycloaddition, quaternary center construction, rhodium

## Abstract

The [RhCl(CO)dppp]_2_-catalyzed intramolecular carbonylative [2 + 2 + 1] cycloaddition of allenenes was developed to prepare bicyclo[4.3.0]nonenones possessing a methyl group at the ring junction, which is difficult to achieve by the Pauson–Khand reaction of the corresponding enynes. This method also provided a new procedure for the construction of the tricyclo[6.4.0.0^1,5^]dodecenone framework in a satisfactory yield.

## Introduction

The Co_2_(CO)_8_-mediated Pauson–Khand reaction (PKR) [1–2] is well recognized as a formal [2 + 2 + 1] cycloaddition of three components, the alkyne π-bond, the alkene π-bond and carbon monoxide (CO), for producing cyclopentenone derivatives. The intramolecular PKR [3–9] provides a powerful method for the construction of bicyclo[3.3.0]oct-1-en-3-one and bicyclo[4.3.0]non-1(9)-en-8-one frameworks. In contrast to the fact that the 2-alkyl (or phenyl)-1-hepten-6-ynes also efficiently afford 5-alkyl (or phenyl)bicyclo[3.3.0]oct-1-en-3-ones [10–19], the one-methylene homologated substrates furnish the corresponding bicyclo[4.3.0] skeletons with an angular substituent in rather lower yields [20–22].

**Scheme 1:**
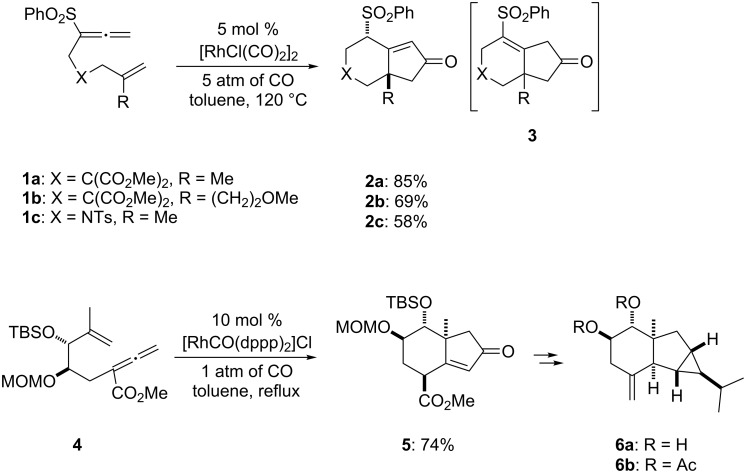
Rh(I)-catalyzed Pauson–Khand type reaction of **1** and **4**.

Recent efforts in our laboratory have developed the [RhCl(CO)_2_]_2_-catalyzed [2 + 2 + 1] cycloaddition of 3-phenylsulfonyl-1,2,7-octatrienes and lead to bicyclo[4.3.0]non-1(9)-en-8-one derivatives, in which the distal double bond of the allenyl moiety serves exclusively as the π-component [23]. This method could not only be applicable for the construction of the larger bicyclo[5.3.0]dec-1(10)-en-9-one skeleton, but also provides a new entry for the preparation of three types of *trans*-2-phenylsulfonylbicyclo[4.3.0]non-1(9)-en-8-one derivatives **2a**–**c** in moderate to high yields with an alkyl group (Me, (CH_2_)_2_OMe) at the ring junction from 7-alkyl-3-phenylsulfonyl-1,2,7-octatrienes **1a**–**c**. The formation of **2** can be rationalized by the initial formation of the 6-alkyl-2-phenylsulfonylbicyclo[4.3.0]non-1-en-8-one derivative **3**, followed by subsequent isomerization to the α,β-unsaturated ketones **2**. By taking advangage of this newly developed reaction, we have completed the first total syntheses of two tricyclic sesquiterpenes **6a**,**b**, isolated from *Jatropha neopauciflora*, from the methoxycarbonylallenene **4** via the bicyclo[4.3.0]nonenone derivative **5** [24]. The key step in these total syntheses was carried out as follows: The allenene **4**, derived from D-tartrate, was heated under reflux in the presence of 10 mol % of [RhCO(dppp)_2_]Cl [25] under a CO atmosphere to produce exclusively the bicyclo[4.3.0]derivative **5** with the desired stereochemistry in 74% yield (Scheme 1). Our continuous interest in this field prompted us to optimize the reaction conditions in order to make our method more useful. This paper describes the Rh(I)-catalyzed [2 + 2 + 1] cycloaddition of allenenes for the facile preparation of the bicyclo[4.3.0]nonenone derivatives possessing an angular methyl group, and for the construction of the tricyclo[6.4.0.0^1,5^]dodecenone skeleton.

## Results and Discussion

In previous studies, we investigated the carbonylative [2 + 2 + 1] cycloaddition reaction of two allenenes **1** and **4** with either a SO_2_Ph or CO_2_Me substituent on the proximal double bond of the allene group in the presence of several Rh(I) catalysts ([RhCl(CO)_2_]_2_, [RhCO(dppp)_2_]Cl or [RhCl(CO)dppp]_2_). We have now examined the similar ring-closing reaction using an alternative allenene **7a** with a methyl group on the allenyl group for further evaluation of these reactions. According to the conditions described for the formation of **2**, a solution of **7a** in toluene was heated at 120 °C for 4 h in the presence of 5 mol % of [RhCl(CO)_2_]_2_ under 5 atm of CO to afford the desired 4,4-bis(methoxycarbonyl)-2,6-dimethylbicyclo[4.3.0]non-1-en-8-one (**8a**) in 38% yield (Table 1, entry 1). A similar reaction under 1 atm of CO gave **8a** in a better yield (Table 1, entry 2). These results are in sharp contrast to the previous findings, where increasing the CO pressure from 1 to 5 atm led to an improvement in the chemical yield (see reference [23], Table 1). The alternative condition ([RhCO(dppp)_2_]Cl (10 mol %) under a CO atmosphere) provided the ring-closing product **8a** in 75% yield (Table 1, entry 3). It has already been shown that the Rh(I)-catalyzed Pauson–Khand type reaction (PKTR) of enynes under a low CO pressure [26–27] leads to better results. Thus, the ring-closing reaction of **7a** was performed under an atmosphere consisting of 0.2 atm of CO and 0.8 atm of Ar, or of 0.05 atm of CO and 0.95 atm of Ar to produce **8a** in 71% and 74% yields, respectively (Table 1, entries 4 and 5). Next, the reaction with [RhCl(CO)dppp]_2_ in the presence or absence of a silver salt [27–34] was examined (Table 1, entries 6–13). A significant improvement (**8a**, 93%) was observed when the ring-closing reaction of **7a** was carried out in the presence of 5 mol % of [RhCl(CO)dppp]_2_ and 12 mol % of AgBF_4_ under an atmosphere consisting of 0.05 atm of CO and 0.95 atm of Ar at 120 °C (Table 1, entry 11). Almost all of the reactions gave 2,6-dimethylbicyclo[4.3.0]non-1-en-8-one **8a**, and the α,β-unsaturated ketone **9a** could not be detected except for entry 9. This observation obviously differs from the PKTR products **2** and **5** having an electron withdrawing group instead of a methyl group (Scheme 1).

**Table 1:** Rh(I)-catalyzed PKTR of **7a**.

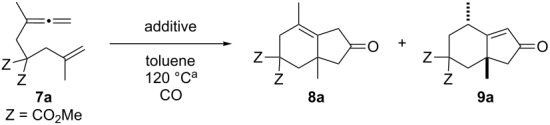					

entry	Rh(I) cat. (mol %)	additive (mol %)	CO (atm)	time (h)	yield of **8a** (%)

1	[RhCl(CO)_2_]_2_ (5)	—	5	4	38
2	[RhCl(CO)_2_]_2_ (5)	—	1	16	54
3	[RhCO(dppp)_2_]Cl (10)	—	1	17	75^b^
4	[RhCO(dppp)_2_]Cl (10)	—	0.2^c^	17	71
5	[RhCO(dppp)_2_]Cl (10)	—	0.05^d^	6	74
6	[RhCl(CO)dppp]_2_ (5)	—	1	19	68
7	[RhCl(CO)dppp]_2_ (5)	—	0.2^c^	11	79
8	[RhCl(CO)dppp]_2_ (5)	—	0.1^e^	7	88
9	[RhCl(CO)dppp]_2_ (5)	—	0.05^d^	8	51^f^
10	[RhCl(CO)dppp]_2_ (5)	AgBF_4_ (12)	0.1^e^	1.5	78
11	[RhCl(CO)dppp]_2_ (5)	AgBF_4_ (12)	0.05^d^	1.5	93
12	[RhCl(CO)dppp]_2_ (5)	AgSbF_6_ (12)	0.05^d^	1.5	79
13	[RhCl(CO)dppp]_2_ (5)	AgCl (12)	0.05^d^	6	84

^a^Bath temperature. ^b^**8a** was obtained in 18% yield under 5 atm of CO. ^c^The reaction was conducted under 0.2 atm of CO and 0.8 atm of Ar. ^d^The reaction was conducted under 0.05 atm of CO and 0.95 atm of Ar. ^e^The reaction was conducted under 0.1 atm of CO and 0.9 atm of Ar. ^f^Compound **9a** was obtained in 31%.

We next investigated the scope of this Rh(I)-catalyzed PKTR using several substrates under the condition of entry 11 in Table 1. The nitrogen congener **7b** produced the corresponding azabicyclic compound **8b** in 56% yield, although a prolonged reaction time was required (Table 2, entry 1). The sulfonylallenene **7c**, which has a simple methylene tether, provided the cyclopentenone derivative **9c** in 55% yield as expected from the results given in Scheme 1 (Table 2, entry 2). As noted above, compounds **2a**,**b** could be obtained in the presence of [RhCl(CO)_2_]_2_: [RhCl(CO)dppp]_2_ also worked as well as the previous conditions (Table 2, entries 3 and 4). The oxygen congener **7d** produced the corresponding oxabicyclic compound **9d** in 49% yield (Table 2, entry 5). The methoxycarbonyl functionality on the allenyl moiety **7e** was found not to interfere with the ring-closing reaction and afforded **9e** in 63% yield (Table 2, entry 6). On the other hand, **7f** provided the oxabicyclic derivative **9f** in a low yield (Table 2, entry 7). The geminal disubstituent effect (Thorpe–Ingold-type effect) of **1a** and **7a** might be responsible for the high yields of the ring-closed products.

**Table 2:** Rh(I)-catalyzed PKTR of 7-methyl-1,2,7-trienes.

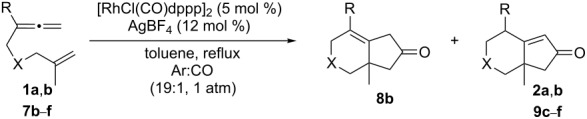				

entry	allenene	product	time (h)	yield (%)

1	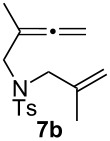	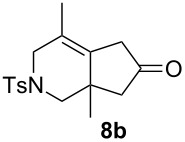	6.5	56
2	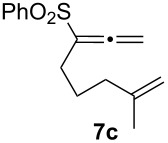	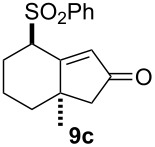	3	55
3	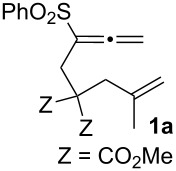	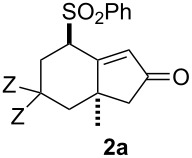	1	88^a,b^
4	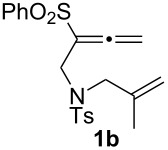	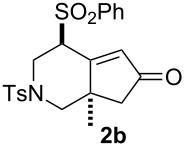	2	66
5	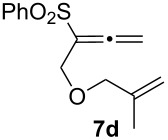	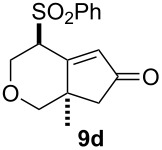	3	49
6	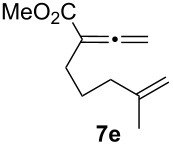	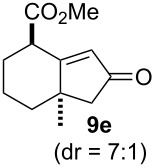	5	63
7	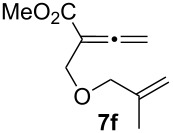	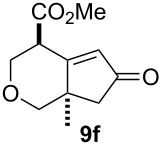	4.5	22

^a^The reaction was carried out without AgBF_4_ under a CO atmosphere. ^b^**2a** was obtained in 68% yield in the presence of 12 mol % of AgBF_4_.

**Scheme 2:**
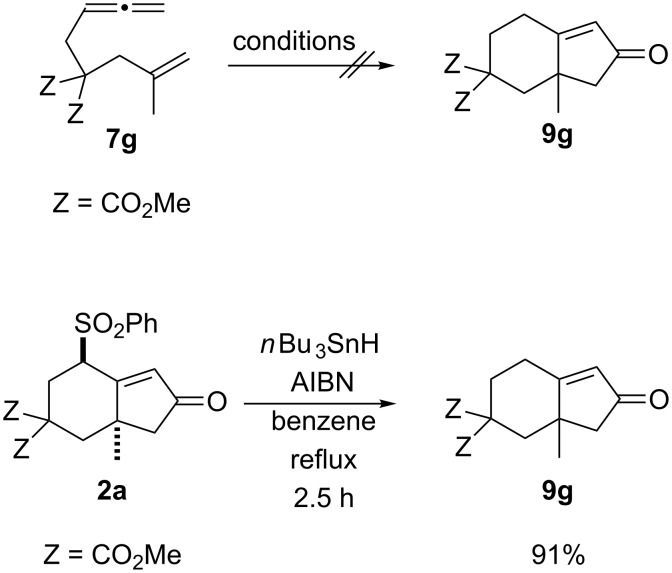
Formation of 4,4-bis(methoxycarbonyl)-6-methylbicyclo[4.3.0]non-1(9)-en-8-one (**9g**).

The Rh(I)-catalyzed PKTR of the non-substituted allenene **7g** was investigated as a control experiment under the several conditions (e.g., conditions of entries 3, 6, 10 and 11 in Table 1) but resulted in the formation of the intractable mixtures (Scheme 2). These results in combination with the successful examples shown in Table 2 indicated that the substituent on the proximal double bond of the allene might be mandatory. Removal of the allylic sulfone group in **2a** was achieved by tributyltin hydride in the presence of AIBN to give **9g** in 91% yield [35–41]. Thus, the phenylsulfonyl group of **2a** can be regarded as a hydrogen surrogate and this procedure provides a new method for the preparation of **9g**.

The intramolecular Rh(I)-catalyzed PKTR of allenenes is applicable to the more complex substrate as shown in Table 3 [42–44]. Exposure of the cyclopentene **10** to the standard conditions in Table 2 did not lead to the desired tricyclo[6.4.0.0^1,5^]dodec-7-en-6-one **11**, instead the spiro[4.5]deca-1,6-diene derivative **12** was formed exclusively in 87% yield (Table 3, entry 1). After screening several reaction conditions, the tricyclic derivative **11** was obtained in 73% yield along with **12** (24%) when the reaction was carried out in the absence of AgBF_4_ under a CO atmosphere (Table 3, entry 2). The [RhCl(CO)dppp]_2_-catalyzed reaction of **10** under a N_2_ atmosphere with or without AgBF_4_ produced the spiro product **12** in 93% and 91% yield, respectively, whereas no spiro product **12** was obtained under thermal conditions without a Rh(I)-catalyst (Table 3, entries 3–5). Therefore, the formation of **12** must be rationalized by the Rh(I)-catalyzed cycloisomerization. The oxidative addition of the Rh(I)-catalyst to an alkene group and the distal double bond of the allenyl moiety would form a rhodabicyclo[4.3.0]nonene intermediate, which would collapse to the spiro[4.5]deca-1,6-diene system via β-elimination and subsequent reductive elimination [31–32].

**Table 3:** Rh(I)-catalyzed cyclization of 6-(cyclopent-1-enyl)-1,2-hexadiene **10**.

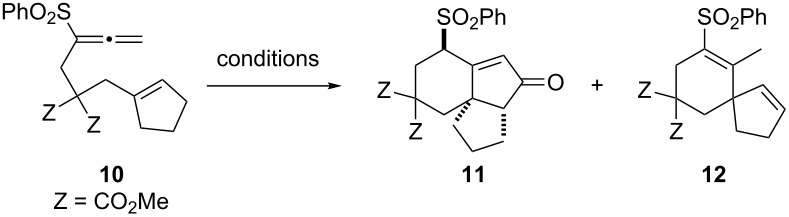					

entry	Rh(I) cat. (5 mol %)	additive (mol %)	CO or N_2_ (atm)	yield of **11** (%)	yield of **12** (%)

1	[RhCl(CO)dppp]_2_	AgBF_4_ (12)	CO (0.05)^a^	—	87
2	[RhCl(CO)dppp]_2_	—	CO (1)	73	24
3	[RhCl(CO)dppp]_2_	AgBF_4_ (12)	N_2_ (1)	—	93
4	[RhCl(CO)dppp]_2_	—	N_2_ (1)	—	91
5	—	—	N_2_ (1)	—	—

^a^The reaction was conducted under 0.05 atm of CO and 0.95 atm of Ar.

In summary, we have developed the intramolecular Rh(I)-catalyzed PKTR between 1,1-disubstituted allene and 1,1-disubstituted alkene functionalities, which leads to the facile formation of bicyclo[4.3.0]nonenone derivatives with an angular methyl group. This method was also successfully applied to the construction of the more complex tricyclo[6.4.0.0^1,5^]dodecenone skeleton.

## Supporting Information

Full experimental details, compound characterization data, ^1^H NMR spectra and ^13^C NMR spectra for all new compounds described.

File 1 Experimental, characterization data and spectra.
