# Supplementary material for: Rh(I)-catalyzed intramolecular [2 + 2 + 1] cycloaddition of allenenes: Construction of bicyclo[4.3.0]nonenones with an angular methyl group and tricyclo[6.4.0.01,5]dodecenone
Source: Beilstein J Org Chem. 2011 Apr 7;7:404–9. doi: 10.3762/bjoc.7.52 (PMC3079109; doi:10.3762/bjoc.7.52)
Supplement: File 1 — Experimental, characterization data and spectra. [file Beilstein_J_Org_Chem-07-404-s001.pdf]

Supporting Information

for

Rh(I)-catalyzed intramolecular [2 + 2 + 1] cycloaddition of allenenes: Construction of bicyclo[4.3.0]nonenones with an angular methyl group and tricyclo[6.4.0.0^{1,5}]dodecenone

Fuyuhiko Inagaki, Naoya Itoh, Yujiro Hayashi, Yumi Matsui and Chisato Mukai*

Address: Division of Pharmaceutical Sciences, Graduate School of Natural Science and Technology, Kanazawa University, Kakuma-machi, Kanazawa 920-1192, Japan

Email: Chisato Mukai - cmukai@kenroku.kanazawa-u.ac.jp
* Corresponding author

Experimental, characterization data and spectra

Table of contents

General S2
Preparation and characterization data for compounds **7a**–**f** S2–7
Reference for compounds **1a**, **1b**, **7g**, **13**, **15**, **17** and **19** S7
Preparation and characterization data for compounds **8a**,**b** and **9a**–**f** S8–10
Reference for compounds **2a**,**b** S10
Preparation and characterization data for compound **9g**, **24** and **10** S11–12
Reference for compounds **23**, **25** S13
Preparation and characterization data for compounds **11**, **12** S13
^{1}H NMR spectra and ^{13}C NMR spectra for compounds **7a**–**f** S14–25
^{1}H NMR spectra and ^{13}C NMR spectra for compounds **8a**,**b** and **9a**–**g** S26–41
^{1}H NMR spectra and ^{13}C NMR spectra for compounds **24**, **10**, **11** and **12** S42–49

General. Melting points are uncorrected. IR spectra were measured in $CHCl_3$. ^1H NMR spectra were taken in $CDCl_3$ expect for the compound **9e** ($(CD_3)_2CO$). $CHCl_3$ (7.26 ppm) for silyl compounds and tetramethylsilane (0.00 ppm) for compounds without a silyl group were used as internal standards unless otherwise stated. ^{13}C NMR spectra were recorded in $CDCl_3$ with $CDCl_3$ (77.00 ppm) as an internal standard unless otherwise stated. All reactions were carried out under a nitrogen atmosphere. Silica gel (silica gel 60, 230–400 mesh) was used for chromatography. Organic extracts were dried over anhydrous Na_2SO_4.

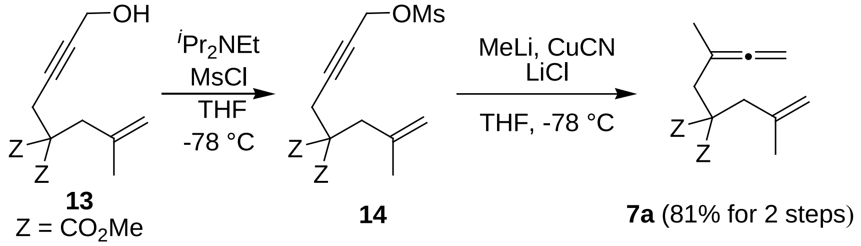

5,5-Bis(methoxycarbonyl)-3,7-dimethylocta-1,2,7-triene (7a).

To a solution of **13** [1] (727 mg, 2.86 mmol) in THF (29 mL) were added $^iPr_2NEt$ (2.0 mL, 11 mmol) and MsCl (0.65 mL, 8.4 mmol) at -78 °C. After stirring for 1.5 h at the same temperature, the reaction mixture was quenched by the addition of saturated aqueous $NaHCO_3$, and the mixture extracted with AcOEt. The extract was washed successively with water and brine, dried and concentrated to dryness. The residue was passed through a short pad of silica gel with hexane-AcOEt (4:1) to afford crude **14**. MeLi (1.09 M in Et_2O, 9.9 mL, 11 mmol) was added gradually to a solution of CuCN (967 mg, 10.8 mmol) and LiCl (915 mg, 21.6 mmol) in THF (27 mL) at -78 °C. The reaction mixture was then warmed to -20 °C and at this temperature the solids dissolved. The reaction mixture was re-cooled to -78 °C and the crude **14** added. After stirring for 1.5 h at the same temperature, the reaction mixture was quenched by the addition of saturated aqueous NH_4Cl and then extracted with AcOEt. The extract was washed successively with water and brine, dried, and concentrated to dryness. The residue was purified by chromatography with hexane-AcOEt (15:1) as eluent to afford **7a** (577 mg, 81% for 2 steps) as a colorless oil: IR 1959, 1732 cm^{-1}; ^1H NMR δ 4.86–4.85 (m, 1H), 4.72–4.71 (m, 1H), 4.59 (sex, 2H, J = 3.0 Hz), 3.69 (s, 6H), 2.81 (s, 2H), 2.62 (t, 2H, J = 3.0 Hz), 1.65–1.64 (m, 6H); ^{13}C NMR δ 206.8, 171.5, 140.7, 115.5, 93.8, 75.2, 56.8, 52.3, 39.8, 35.1, 23.3, 20.3; MS m/z 252 (M^+, 31.2); HRMS calcd for $C_{14}H_{20}O_4$ 252.1362, found 252.1363.

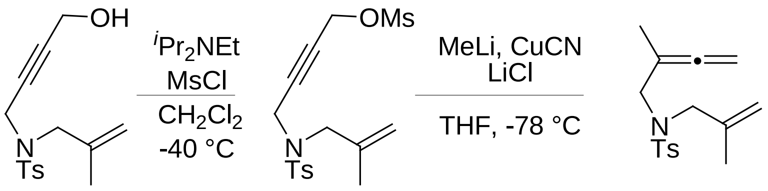

15 **16** **7b** (56% for 2 steps)

3,7-Dimethyl-*N*-(4-methylbenzenesulfonyl)-5-azaocta-1,2,7-triene (7b).

To a solution of **15** [2] (100 mg, 0.340 mmol) in CH_2Cl_2 (3.4 mL) were added ^{i}Pr$_2$NEt (0.24 mL, 1.4 mmol) and MsCl (0.080 mL, 1.0 mmol) at -40 °C. After stirring for 1 h at the same temperature, the reaction mixture was quenched by the addition of saturated aqueous $NaHCO_3$ and the mixture extracted with CH_2Cl_2. The extract was washed successively with water and brine, dried and concentrated to dryness. The residue was passed through a short pad of silica gel with hexane-AcOEt (2:1) as eluent to afford crude **16**. MeLi (1.09 M in Et_2O, 1.20 mL, 1.36 mmol) was added gradually to a solution of CuCN (121 mg, 1.36 mmol) and LiCl (115 mg, 2.72 mmol) in THF (3.4 mL) at -78 °C. The reaction mixture was then warmed to -20 °C and at this temperature the solids dissolved. The reaction mixture was re-cooled to -78 °C and the crude **16** added. After stirring for 1.5 h at the same temperature, the reaction mixture was quenched by the addition of saturated aqueous NH_4Cl and extracted with AcOEt. The extract was washed successively with water and brine, dried and concentrated to dryness. The residue was purified by chromatography with hexane-AcOEt (15:1) as eluent to afford **7b** (56.0 mg, 56% for 2 steps) as a yellow oil: IR 1961, 1338, 1157 cm^{-1}; ^{1}H NMR δ 7.69 (d, 2H, *J* = 8.2 Hz), 7.28 (d, 2H, *J* = 8.2 Hz), 4.87 (s, 1H), 4.81 (s, 1H), 4.48 (sex, 2H, *J* = 3.0 Hz), 3.739 (t, 2H, *J* = 3.0 Hz), 3.731 (s, 2H), 2.41 (s, 3H), 1.63 (s, 3H), 1.57 (t, 3H, *J* = 3.0 Hz); ^{13}C NMR δ 207.3, 142.9, 140.0, 137.5, 129.4, 127.1, 114.2, 94.2, 74.9, 53.2, 50.3, 21.4, 19.9, 16.1; MS *m/z* 291 (M$^+$, 20.4); HRMS calcd for $C_{16}H_{21}NO_2S$ 291.1293, found 291.1292.

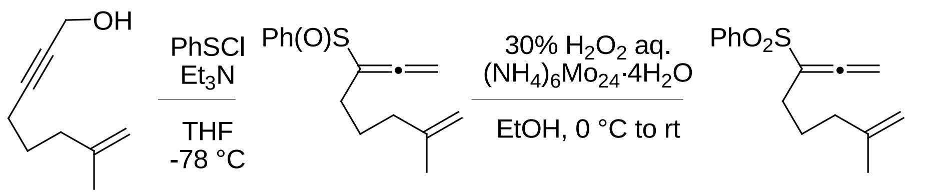

7-Methyl-3-phenylsulfonylocta-1,2,7-triene (7c).

PhSCl (310 mg, 2.2 mmol) in THF (2.2 mL) was added gradually to a solution of **17** [2] (100 mg, 0.724 mmol) and Et_3N (0.90 mL, 6.5 mmol) in THF (4.8 mL) at -78 °C. After stirring for 2.5 h at the same temperature, the reaction was quenched by the addition of saturated aqueous $NaHCO_3$ and the mixture extracted with AcOEt. The extract was washed successively with water and brine, dried and concentrated to dryness. The residue was passed through a short pad of silica gel with hexane-AcOEt (9:1) as eluent to afford crude **18**. To a solution of $(NH_4)_6Mo_7O_{24}\cdot 4H_2O$ (520 mg, 0.421 mmol) in EtOH (4.0mL) was added 30% H_2O_2 (2.0 mL) at 0 °C. After stirring for 30 min at the same temperature, the crude **18** in EtOH (8.0 mL) was added dropwise to the reaction mixture at the same temperature. After the complete addition of the crude **18**, the reaction mixture was warmed to room temperature and stirred overnight. The reaction was quenched by the addition of saturated aqueous $NaHCO_3$ and then extracted with AcOEt. The extract was washed successively with water and brine, dried and concentrated to dryness. The residue was purified by chromatography with hexane-AcOEt (15:1) as eluent to afford **7c** (44.8 mg, 24% for 2 steps) as a pale yellow oil: IR 1969, 1940, 1649, 1317, 1307, 1149 cm^{-1}; ^{1}H NMR δ 7.89 (d, 2H, J = 7.8 Hz), 7.62 (t, 1H, J = 7.8 Hz), 7.53 (t, 2H, J = 7.8 Hz), 5.36 (t, 2H, J = 3.4 Hz), 4.66 (s, 1H), 4.57 (s, 1H), 2.22 (tt, 2H, J = 7.5, 3.4 Hz), 1.96 (t, 2H, J = 7.5 Hz), 1.63 (s, 3H), 1.56 (quin, 2H, J = 7.5 Hz); ^{13}C NMR δ 207.7, 144.6, 140.1, 133.4, 129.0, 128.0, 113.1, 110.5, 84.3, 36.7, 26.1, 25.3, 22.1; MS *m/z* 262 (M^+, 7.0); HRMS calcd for $C_{15}H_{18}O_2S$ 262.1028, found 262.1023.

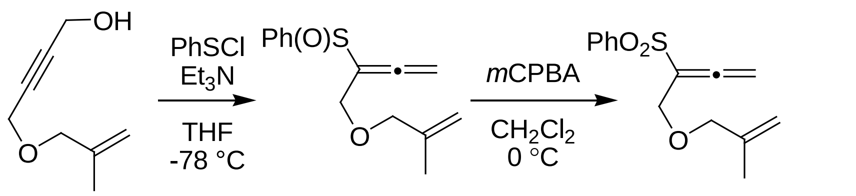

19 **20** **7d** (76% for 2 steps)

7-Methyl-3-phenylsulfonyl-5-oxaocta-1,2,7-triene (7d).

PhSCl (430 mg, 3.0 mmol) in THF (3.0 mL) was added gradually to a solution of **19** [3] (140 mg, 1.00 mmol) and Et_3N (0.42 mL, 3.0 mmol) in THF (10 mL) at -78 °C. After stirring for 2.5 h at the same temperature, the reaction was quenched by the addition of saturated aqueous $NaHCO_3$ and the mixture extracted with AcOEt. The extract was washed successively with water and brine, dried, and concentrated to dryness. The residue was passed through a short pad of silica gel with hexane-AcOEt (4:1) as eluent to afford the crude sulfoxide. To a solution of the crude sulfoxide in CH_2Cl_2 (10 mL) was added *m*CPBA (313 mg, 1.20 mmol) at the 0 °C. After stirring for 1 h, the reaction mixture was quenched by the addition of saturated aqueous $Na_2S_2O_3$ and $NaHCO_3$ and then extracted with CH_2Cl_2. The extract was washed successively with water and brine, dried and concentrated to dryness. The residue was purified by chromatography with hexane-AcOEt (10:1) as eluent to afford **7d** (200 mg, 76% for 2 steps) as a colorless oil: IR 1967, 1929, 1321, 1153 cm^{-1}; ^1H NMR δ 7.94–7.91 (m, 2H), 7.63–7.58 (m, 1H), 7.54–7.50 (m, 2H), 5.47 (d, 1H, J = 2.0 Hz), 5.46 (d, 1H, J = 2.0 Hz), 4.81 (s, 1H), 4.78 (s, 1H), 4.28 (d, 1H, J = 2.1 Hz), 4.27 (d, 1H, J = 2.1 Hz), 3.69 (s, 2H), 1.60 (s, 3H); ^{13}C NMR δ 209.4, 140.9, 140.8, 133.3, 128.8, 127.8, 112.7, 110.1, 83.6, 73.6, 65.4, 19.1; MS *m/z* 264 (M^+, 1.6); HRMS calcd for $C_{14}H_{16}O_3S$ 264.0820, found 264.0817.

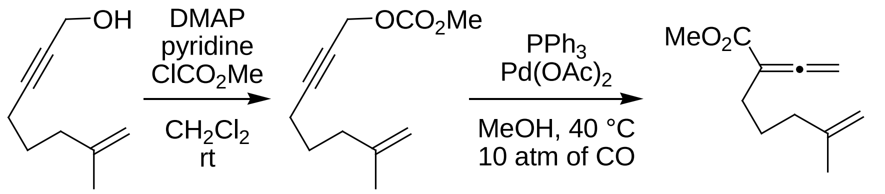

17 **21** **7e** (38% for 2 steps)

3-Methoxycarbonyl-7-methylocta-1,2,7-triene (7e).

To a solution of **17** [2] (100 mg, 0.724 mmol) in CH_2Cl_2 (3.4 mL) were added DMAP (18 mg, 0.15 mmol), pyridine (0.63 mL, 7.8 mmol) and $ClCO_2Me$ (0.20 mL, 2.6 mmol) at room temperature. After stirring for 5 h at the same temperature, the reaction mixture was quenched by the addition of saturated aqueous $NaHCO_3$ and extracted with CH_2Cl_2. The extract was washed successively with water, 10% aqueous HCl and brine, dried, and concentrated to dryness. The residue was passed through a short pad of silica gel with hexane-AcOEt (10:1) as eluent to afford crude **21**. To a solution of the crude **21** in MeOH (1.0 mL) were added $Pd(OAc)_2$ (5.8 mg, 2.6 x 10^{-2} mmol) and PPh_3 (27.3 mg, 0.104 mmol) at room temperature. The reaction mixture was warmed to 40 °C under CO (10 atm) and stirred for 12 h. The MeOH was evaporated and the residue purified by chromatography with hexane-AcOEt (20:1) as eluent to afford **7e** (50.0 mg, 38% for 2 steps) as a colorless oil: IR 1967, 1936, 1712 cm^{-1}; ^1H NMR δ 5.09 (t, 2H, J = 3.0 Hz), 4.67–4.63 (m, 2H), 3.70 (s, 3H), 2.19 (tt, 2H, J = 7.7, 3.0 Hz), 2.01 (t, 2H, J = 7.7 Hz), 1.67 (s, 3H), 1.56 (quin, 2H, J = 7.7 Hz); ^{13}C NMR δ 213.8, 167.6, 145.3, 110.1, 99.9, 78.9, 52.1, 37.0, 27.5, 25.8, 22.2; MS *m/z* 180 (M^+, 8.2); HRMS calcd for $C_{11}H_{16}O_2$ 180.1150, found 180.1146.

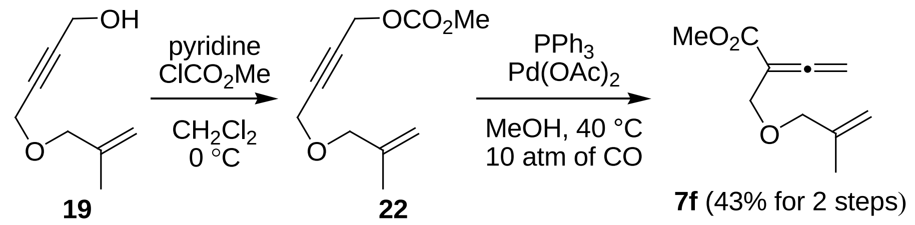

3-Methoxycarbonyl-7-methyl-5-oxaocta-1,2,7-triene (7f).

To a solution of **19** [3] (142 mg, 1.00 mmol) in CH_2Cl_2 (5.0 mL) were added pyridine (0.24 mL, 3.0 mmol) and $ClCO_2Me$ (0.15 mL, 2.0 mmol) at 0 ˚C. After stirring for 30 min at the same temperature, the reaction mixture was quenched by the addition of water and extracted with CH_2Cl_2. The extract was washed successively with water, 5% aqueous HCl and brine, dried, and concentrated to dryness. The residue was passed through a short pad of silica gel with hexane-AcOEt (5:1) as eluent to afford crude **22** (194 mg). To a solution of the crude **22** (105 mg) in MeOH (1.0 mL) were added $Pd(OAc)_2$ (5.7 mg, 2.5 x 10^{-2} mmol) and PPh_3 (26.2 mg, 0.100 mmol) at room temperature. The reaction mixture was warmed to 40 °C under CO (10 atm) and stirred for 11 h. The MeOH was evaporated and the residue purified by chromatography with hexane-AcOEt (50:1) as eluent to afford **7f** (42.0 mg, 43% for 2 steps) as a colorless oil: IR 1967, 1930, 1751, 1713 cm^{-1}; ^{1}H NMR δ 5.22 (d, 1H, *J* = 2.1 Hz), 5.21 (d, 1H, *J* = 2.1 Hz), 4.97–4.91 (m, 1H), 4.89–4.84 (m, 1H), 4.15 (d, 1H, *J* = 2.1 Hz), 4.14 (d, 1H, *J* = 2.1 Hz), 3.90 (s, 2H), 3.73 (s, 3H), 1.70 (s, 3H); ^{13}C NMR δ 214.6, 166.3, 141.8, 112.7, 97.9, 79.6, 74.3, 66.4, 52.3, 19.4; FABMS *m/z* 183 (M^++1 , 10.9); FABHRMS calcd for $C_{10}H_{15}O_3$ 183.1021, found 183.1016.

Alcohols **13**, **15**, **17**, **19** were known compounds, see: [1] Kitamura, T.; Sato, Y.; Mori, M. *Adv. Synth. Catal.* **2002**, *344*, 678–693; [2] Shen, K.-H.; Lush, S.-F.; Chen, T.-L.; Liu, R.-S. *J. Org. Chem.* **2001**, *66*, 8106–8111; [3] Padwa, A.; Lipka, H.; Watterson, S. H.; Murphree, S. S. *J. Org. Chem.* **2003**, *68*, 6238–6250.

Allenenes **1a**, **1b**, **7g** were known compounds, see: [4] Inagaki, F.; Mukai, C. *Org. Lett.* **2006**, *8*, 1217–1220; [5] Tarselli, M. A.; Chianese, A. R.; Lee, S. J.; Gagné, M. R. *Angew. Chem., Int. Ed.* **2007**, *46*, 6670–6673.

General Procedure for Pauson–Khand-type [2 + 2 + 1] cycloaddition under the atmosphere consisting of 0.05 atm of CO and 0.95 atm of Ar.

To a solution of allenene (0.100 mmol) in toluene (1.0 mL) were added 5 mol % $[RhCl(CO)dppp]_2$ and 12 mol % $AgBF_4$ in toluene (0.2 mL) and then stirred for 15 min at room temperature under an argon atmosphere. The argon atmosphere was replaced with the atmosphere consisting of CO and argon (1:19) and the reaction mixture was heated under reflux until complete disappearance of the starting material (monitored by TLC). The toluene was evaporated and the residual oil purified by chromatography with hexane-AcOEt as eluent to afford the cyclized product. Chemical yields are summarized in Table 1 and 2.

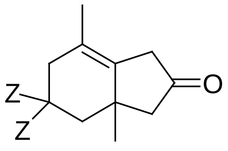

8a
Z = CO_2Me

4,4-Bis(methoxycarbonyl)-2,6-dimethylbicyclo[4.3.0]non-1-en-8-one (8a).

Compound **8a**, colorless oil; IR 1747, 1732 cm^{-1}; ^1H NMR δ 3.71 (s, 3H), 3.70 (s, 3H), 2.96–2.83 (m, 3H), 2.41 (d, 1H, *J* = 14.0 Hz), 2.31–2.20 (m, 4H), 1.66 (s, 3H), 0.97 (s, 3H); ^{13}C NMR δ 215.5, 172.2, 172.1, 132.4, 124.2, 56.6, 53.0, 52.7, 52.5, 39.8, 39.6, 39.2, 35.2, 26.1, 19.6; MS *m/z* 280 (M^+, 51.8); HRMS calcd for $C_{15}H_{20}O_5$ 280.1310, found 280.1307.

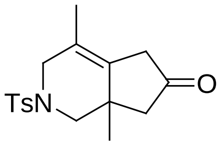

8b

2,6-Dimethyl-*N*-(4-methylphenylsulfonyl)-4-azabicyclo[4.3.0]non-1-en-8-one (8b).

Compound **8b**, yellow oil; IR 1747, 1338, 1166 cm^{-1}; ^1H NMR δ 7.68 (d, 2H, *J* = 8.5 Hz), 7.33 (d, 2H, *J* = 8.5 Hz), 3.96 (d, 1H, *J* = 16.4 Hz), 3.85 (d, 1H, *J* = 10.3 Hz), 3.04 (d, 1H, *J* = 16.4 Hz), 2.89 (s, 2H), 2.43 (s, 3H), 2.31 (d, 1H, *J* = 10.3 Hz), 2.25 (d, 1H, *J* = 16.4 Hz), 2.05 (d, 1H, *J* = 16.4 Hz), 1.57 (s, 3H), 1.27 (s, 3H); ^{13}C NMR δ 213.9, 143.6, 133.5, 132.6, 129.7, 127.5, 122.3, 53.8, 51.6, 48.4, 40.7, 39.1, 24.3, 21.4, 16.6; MS *m/z* 319 (M^+, 53.4); HRMS calcd for $C_{17}H_{21}NO_3S$ 319.1242, found 319.1238.

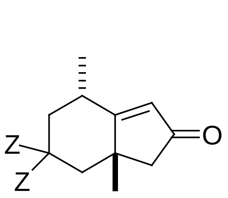

9a
Z = CO_2Me

(2*R,6*R**)-4,4-Bis(methoxycarbonyl)-2,6-dimethylbicyclo[4.3.0]non-1(9)-en-8-one (*trans*-9a).**

Compound **9a**, colorless oil; IR 1730, 1685, 1616 cm^{-1}; ^1H NMR δ 5.81 (d, 1H, *J* = 1.7 Hz), 3.78 (s, 3H), 3.70 (s, 3H), 2.98 (dquin, 1H, *J* = 12.6, 6.3 Hz), 2.70–2.66 (m, 2H), 2.30 (s, 2H), 2.05 (d, 1H, *J* = 14.4 Hz), 1.33–1.28 (m, 1H), 1.21 (d, 3H, *J* = 6.3 Hz), 1.11 (s, 3H); ^{13}C NMR δ 206.6, 189.7, 171.9, 171.4, 124.9,

53.6, 53.2, 53.0, 52.6, 43.5, 42.8, 40.4, 28.9, 25.4, 17.8; MS *m/z* 280 (M^+, 51.8); HRMS calcd for $C_{15}H_{20}O_5$ 280.1310, found 280.1307.

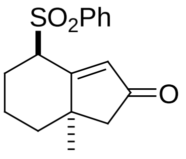

9c

(2*R,6*R**)-6-Methyl-2-(phenylsulfonyl)bicyclo[4.3.0]non-1(9)-en-8-one (*trans*-9c).**

Compound **9c**, colorless powder; mp 224–226 °C (hexane-AcOEt); IR 1704, 1616, 1309, 1145 cm^{-1}; ^1H NMR δ 7.93 (d, 2H, *J* = 7.5 Hz), 7.69 (t, 1H, *J* = 7.5 Hz), 7.60 (t, 2H, *J* = 7.5 Hz), 6.50 (s, 1H), 4.03 (dd, 1H, *J* = 13.0, 4.0 Hz), 2.29 (d, 1H, *J* = 18.5 Hz), 2.23 (d, 1H, *J* = 18.5 Hz), 2.19–2.17 (m, 1H), 1.93 (d, 1H, *J* = 13.0 Hz), 1.83–1.79 (m, 1H), 1.75 (td, 1H, *J* = 13.0, 4.0 Hz), 1.70–1.64 (m, 1H), 1.44 (td, 1H, *J* = 13.0, 4.0 Hz), 1.21 (s, 3H); ^{13}C NMR δ 206.3, 173.8, 137.6, 134.1, 129.4, 128.6, 128.5, 62.4, 50.8, 44.7, 39.8, 28.6, 23.7, 20.9; MS *m/z* 290 (M^+, 0.7); HRMS calcd for $C_{16}H_{18}O_3S$ 290.0977, found 290.0976.

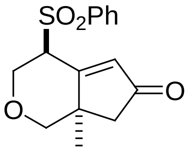

9d

(2*R,6*S**)-6-Methyl-2-(phenylsulfonyl)-4-oxabicyclo[4.3.0]non-1(9)-en-8-one (*trans*-9d).**

Compound **9d**, colorless plates; mp 141–143 °C (hexane-AcOEt); IR 1717, 1618, 1327, 1151 cm^{-1}; ^1H NMR δ 7.96–7.92 (m, 2H), 7.78–7.71 (m, 1H), 7.66–7.62 (m, 2H), 6.61 (d, 1H, *J* = 1.5 Hz), 4.32 (ddd, 1H, *J* = 10.7, 5.4, 1.5 Hz), 4.20 (dd, 1H, *J* = 10.7, 5.4 Hz), 3.89 (d, 1H, *J* = 11.0 Hz), 3.62 (t, 1H, *J* = 10.7 Hz), 3.26 (d, 1H, *J* = 11.0 Hz), 2.21 (d, 1H, *J* = 18.7 Hz), 2.13 (d, 1H, *J* = 18.7 Hz), 1.34 (s, 3H); ^{13}C NMR δ 204.9, 170.3, 137.6, 134.7, 129.7, 128.7, 128.4, 78.0, 68.3, 61.2, 45.2, 45.1, 23.2; MS *m/z* 292 (M^+, 5.6); HRMS calcd for $C_{15}H_{16}O_4S$ 292.0769, found 292.0767.

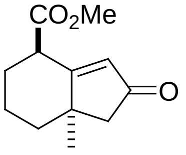

***trans*-9e**

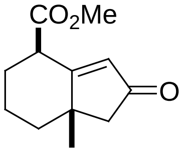

***cis*-9e**

(2*R,6*R**)-and (2*R**,6*S**)-2-Methoxycarbonyl-6-methylbicyclo[4.3.0]non-1(9)-en-8-one (*trans*-9e and *cis*-9e).**

A mixture of *trans*-**9e** and *cis*-**9e** in the ratio of 87:13 was obtained as a colorless oil; IR 1737, 1701, 1616 cm^{-1}; ^1H NMR δ 5.92 (s, 13/100 x 1H), 5.64 (d, 87/100 x 1H, *J* = 1.4 Hz), 3.88 (d, 13/100 x 1H, *J* = 5.1 Hz), 3.72 (s, 87/100 x 3H), 3.65 (s, 13/100 x 3H), 3.59 (ddd, 87/100 x 1H, *J* = 12.1, 5.1, 1.4 Hz), 2.45–2.36 (m, 13/100 x 1H), 2.19–2.11 (m, 87/100 x 2H, 13/100 x 2H), 2.03 (quin, 87/100 x 1H, *J* = 2.3 Hz), 1.98–1.70 (m, 87/100 x 3H, 13/100 x 1H), 1.69–1.58 (m, 87/100 x

1H, 13/100 x 1H), 1.55–1.52 (m, 13/100 x 2H), 1.50–1.40 (m, 87/100 x 1H, 13/100 x 1H), 1.29 (s, 87/100 x 3H), 1.12 (s, 13/100 x 3H); ^{13}C NMR δ 207.3, 206.8, 182.4, 181.9, 172.3, 171.4, 130.4, 126.2, 52.6, 52.2, 52.0, 51.8, 44.1, 43.58, 43.53, 43.3, 40.4, 40.1, 30.5, 28.4, 24.3, 24.1, 20.8, 18.9; MS *m/z* 208 (M^+, 38.5); HRMS calcd for $C_{12}H_{16}O_3$ 208.1099, found 208.1103.

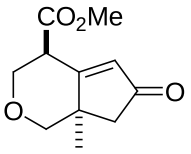

9f

(2*R,6*S**)-2-Methoxycarbonyl-6-methyl-4-oxabicyclo[4.3.0]non-1(9)-en-8-one (*trans*-9f).**

Compound **9f**, colorless oil; IR 1738, 1713, 1620 cm^{-1}; ^{1}H NMR δ 5.96 (d, 1H, *J* = 1.8 Hz), 4.37 (dd, 1H, *J* = 11.0, 5.5 Hz), 3.92 (d, 1H, *J* = 11.0 Hz), 3.80 (ddd, 1H, *J* = 11.0, 5.5, 1.8 Hz), 3.77 (s, 3H), 3.54 (t, 1H, *J* = 11.0 Hz), 3.25 (d, 1H, *J* = 11.0 Hz), 2.24 (d, 1H, *J* = 18.5 Hz), 2.13 (d, 1H, *J* = 18.5 Hz), 1.40 (s, 3H); ^{13}C NMR δ 205.4, 178.2, 169.6, 126.7, 78.3, 69.9, 52.2, 46.1, 44.2, 44.0, 23.2; MS *m/z* 210 (M^+, 100.0); HRMS calcd for $C_{11}H_{14}O_4$ 210.0892, found 210.0894.

Synthesis of (2*R,6*R**)-4,4-Bis(methoxycarbonyl)-6-methyl-2-phenylsulfonylbicyclo[4.3.0]non-1(9)-en-8-one (*trans*-2a).**

To a solution of **1a** (37.2 mg, 0.10 mmol) in toluene (1.0 mL) was added $[RhCl(CO)dppp]_2$ (6.0 mg, 5.0 x 10^{-3} mmol). The reaction mixture was heated under reflux for 1 h under CO (1 atm). The solvent was evaporated and the residue purified by chromatography with hexane-AcOEt (1:1) as eluent to afford **2a** (35.2 mg, 88%).

Bicyclic compounds **2a**, **2b** were known compounds, see reference [4]: Inagaki, F.; Mukai, C. *Org. Lett.* **2006**, *8*, 1217–1220.

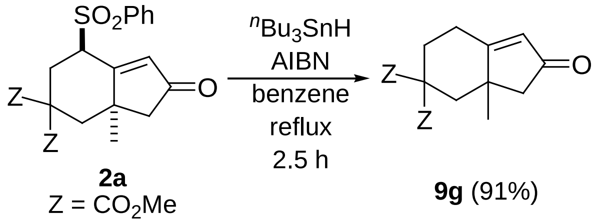

4,4-Bismethoxycarbonyl-6-methyl-bicyclo[4.3.0]non-1(9)-en-8-one (9g).

To a solution of **2a** (40.6 mg, 0.100 mmol) in benzene (2.0 mL) were added ^{n}Bu$_3$SnH (0.05 mL, 0.2 mmol) and AIBN (1.6 mg, 9.7 x 10^{-3} mmol). After heating under reflux for 2.5 h, the solvent was evaporated and the residue purified by chromatography with hexane-AcOEt (10:1) as eluent to afford **9g** (24.3 mg, 91%) as colorless needles, mp 70–72 °C (hexane); IR 1730, 1683, 1625 cm^{-1}; ^{1}H NMR δ 5.83 (d, 1H, *J* = 1.4 Hz), 3.79 (s, 3H), 3.70 (s, 3H), 2.82 (tdd, 1H, *J* = 13.6, 5.0, 1.4 Hz), 2.77–2.72 (m, 1H), 2.70–2.66 (m, 2H), 2.28 (d, 2H, *J* = 2.4 Hz), 2.09 (d, 1H, *J* = 13.6 Hz), 1.64–1.58 (m, 1H), 1.10 (s, 3H); ^{13}C NMR δ 206.8, 185.0, 171.7, 171.5, 127.0, 53.0, 52.9, 52.8, 52.6, 43.4, 42.2, 32.2, 24.9, 24.5; MS *m/z* 266 (M^{+}, 30.8); HRMS calcd for $C_{14}H_{18}O_5$ 266.1154, found 266.1154.

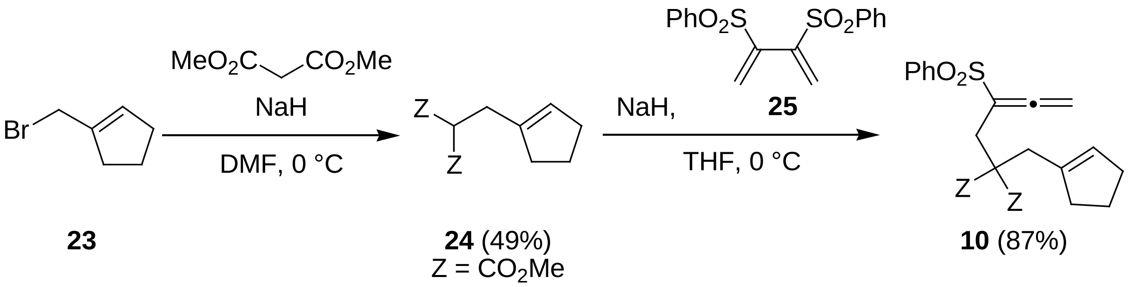

Dimethyl (cyclopent-1-en-1-ylmethyl)malonate (24).

To a solution of dimethyl malonate (32 mg, 0.24 mmol) in DMF (2.0 mL) was added NaH (9.6 mg, 0.24 mmol) at 0 ºC. After stirring for 1 h, **23** [6] (13 mg, 0.080 mmol) in DMF (1.0 mL) was added to the reaction mixture at 0 ºC. After stirring for 2 h at room temperature, the reaction mixture was quenched by addition of water and extracted with Et_2O. The extract was washed successively with water and brine, dried and concentrated to dryness. The residue was purified by chromatography with hexane-AcOEt (4:1) as eluent to afford **24** as a yellow oil (8.4 mg, 49%): IR 1751, 1732 cm^{-1}; ^{1}H NMR δ 5.38 (t, 1H, *J* = 1.3 Hz), 3.72 (s, 6H), 3.60 (t, 1H, *J* = 7.6 Hz), 2.68 (dd, 2H, *J* = 7.6, 1.3 Hz), 2.28–2.21 (m, 4H), 1.85 (m, 2H); ^{13}C NMR δ 169.9, 140.3, 125.9, 52.5, 50.5, 34.9, 32.5, 30.4, 23.3; MS *m/z* 212 (M^{+}, 8.4); HRMS calcd for $C_{11}H_{16}O_4$ 212.1049, found 212.1051.

Dimethyl 2-(cyclopent-1-en-1-ylmethyl)-2-[2-(phenylsulfonyl)buta-2,3-dienyl]malonate (10).

To a solution of **24** (212 mg, 1.00 mmol) in THF (10 mL) was added NaH (40 mg, 1.0 mmol) at 0 ºC. After stirring for 1.5 h, a solution of **25** [7] (334 mg, 1.00 mmol) in THF (10 mL) was added to the reaction mixture at 0 ºC. After stirring for 10 min at the same temperature, the reaction mixture was quenched by addition of water and extracted with AcOEt. The extract was washed successively with water and brine, dried and concentrated to dryness. The residue was purified by chromatography with hexane-AcOEt (3:1 to 1:1) as eluent to afford **10** (353 mg, 87%) as colorless needles: mp 113 ºC (hexane-AcOEt); IR 1971, 1938, 1736, 1225, 1153 cm^{-1}; ^{1}H NMR δ 7.88 (d, 2H, *J* = 7.5 Hz), 7.63 (t, 1H, *J* = 7.5 Hz), 7.55 (t, 2H, *J* = 7.5 Hz), 5.41 (t, 2H, *J* = 3.8 Hz), 5.23 (s, 1H), 3.64 (s, 6H), 2.90 (t, 2H, *J* = 3.8 Hz), 2.81 (s, 2H), 2.16–2.14 (m, 2H), 2.02–2.00 (m, 2H), 1.75–1.70 (m, 2H); ^{13}C NMR δ 207.9, 170.3, 139.9, 137.9, 133.5, 129.8, 129.1, 128.2, 109.4, 85.8, 56.1, 52.6, 35.4, 33.5, 32.3, 28.4, 23.6; MS *m/z* 404 (M^+, 4.2); Anal. Calcd for $C_{21}H_{24}O_6S$: C, 62.36; H, 5.98. Found: C, 62.30; H 6.01.

Compounds **23** and **25** were known compounds, see: [6] Kim, D. D.; Lee, S. J.; Beak, P. *J. Org. Chem.* **2005**, *70,* 5376–5386. [7] Jaganathan, S.; Okamura, W. H. *Tetrahedron Lett.* **1982**, *23,* 4763–4764.

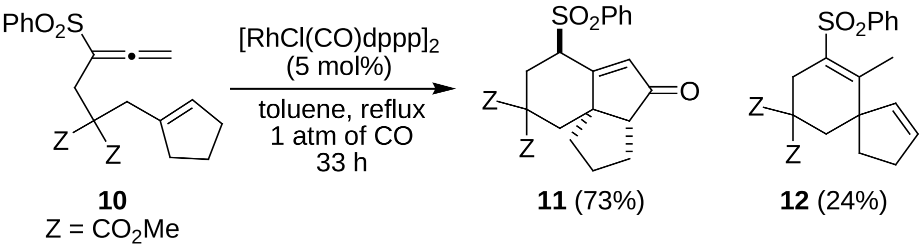

11-Bis(methoxycarbonyl)-9-phenylsulfonyltricyclo[6.4.0.0^{1,5}]dodec-7-ene-6-one (11) and 9-Bismethoxycarbonyl-7-phenylsulfonylspiro[4.5]deca-1,6-diene (12).

According to the same procedure described for preparation of **2a**, **11** (31 mg, 73%) and **12** (9.9 mg, 24%) were obtained from **10** (40 mg, 0.10 mmol).

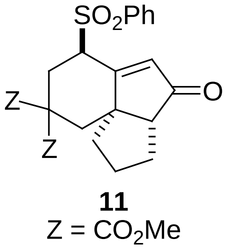

Compound **11**, colorless needles; mp 196–197 ºC (hexane-AcOEt); IR 1732, 1707, 1618 cm^{-1}; ^{1}H NMR δ 7.98 (d, 2H, *J* = 7.3 Hz), 7.72 (t, 1H, *J* = 7.3 Hz), 7.63 (t, 2H, *J* = 7.3 Hz), 6.67 (s, 1H), 4.63–4.60 (m, 1H), 3.72 (s, 3H), 3.70 (s, 3H), 2.67–2.64 (m, 2H), 2.38 (d, 1H, *J* = 9.2 Hz), 2.30 (d, 1H, *J* = 14.0 Hz), 2.00–1.90 (m, 2H), 1.74–1.59 (m, 3H), 1.33–1.18 (m, 2H); ^{13}C NMR δ 209.1, 171.1, 170.0, 169.4, 137.6, 134.4, 130.9, 129.5, 128.5, 59.5, 58.5, 55.5, 53.4, 53.3, 52.9, 43.0, 34.3, 33.0, 28.6, 25.2; MS *m/z* 432 (M$^+$, 11).; Anal. Calcd for $C_{22}H_{24}O_7S$: C, 61.10; H, 5.59. Found: C, 61.02; H, 5.63.

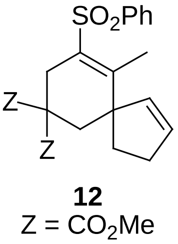

Compound **12**, yellow oil; IR 1734, 1628, 1225, 1157 cm^{-1}; ^{1}H NMR δ 8.02–8.00 (m, 2H), 7.62–7.59 (m, 1H), 7.56–7.54 (m, 2H), 5.86 (dt, 1H, *J* = 5.5, 2.1 Hz), 5.22 (dt, 1H, *J* = 5.5, 2.1 Hz), 3.68 (s, 3H), 3.64 (s, 3H), 3.17 (dt, 1H, *J* = 17.8, 1.4 Hz), 2.83 (dd, 1H, *J* = 17.8, 2.1 Hz), 2.50–2.42 (m, 1H), 2.38–2.31 (m, 1H), 2.22 (d, 2H, *J* = 5.5 Hz), 1.88 (t, 3H, *J* = 1.4 Hz), 1.82–1.76 (m, 1H), 1.64–1.58 (m, 1H); ^{13}C NMR δ 171.2, 170.9, 151.4, 141.8, 136.0, 133.1, 132,9, 131.6, 128.9, 127.1, 55.4, 52.7, 52.5, 51.7, 39.7, 34.9, 32.7, 32.1, 15.8; MS *m/z* 404 (M$^+$, 10.4); HRMS calcd for $C_{21}H_{24}O_6S$ 404.1294, found 404.1298.

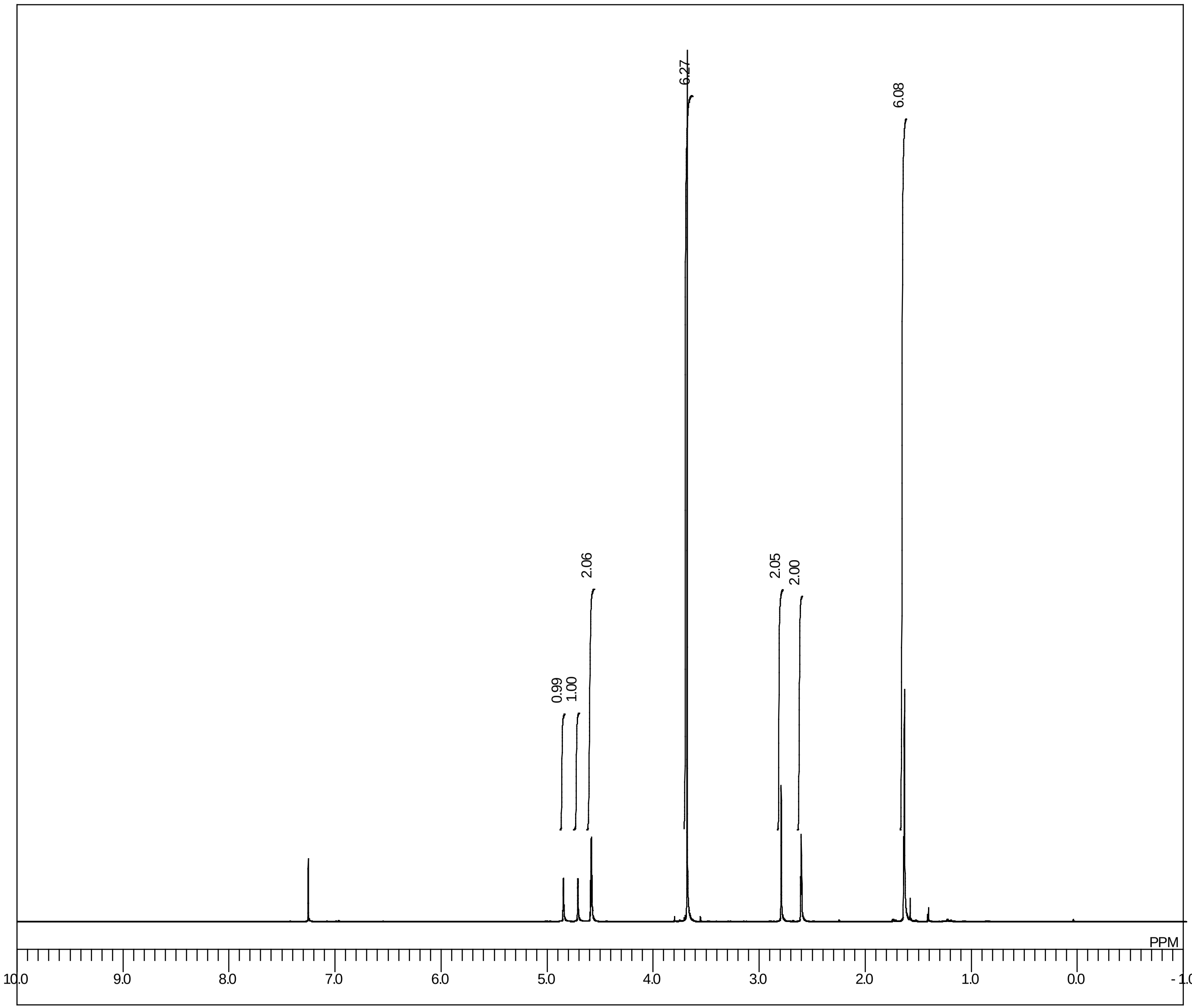

OBNUC 1H
EXMOD single_pulse.ex2
OBFRQ 600.17 MHz
SLVNT CDCL3
EXREF 7.26 ppm

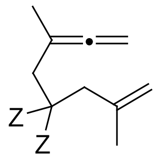

7a
Z = CO_2Me

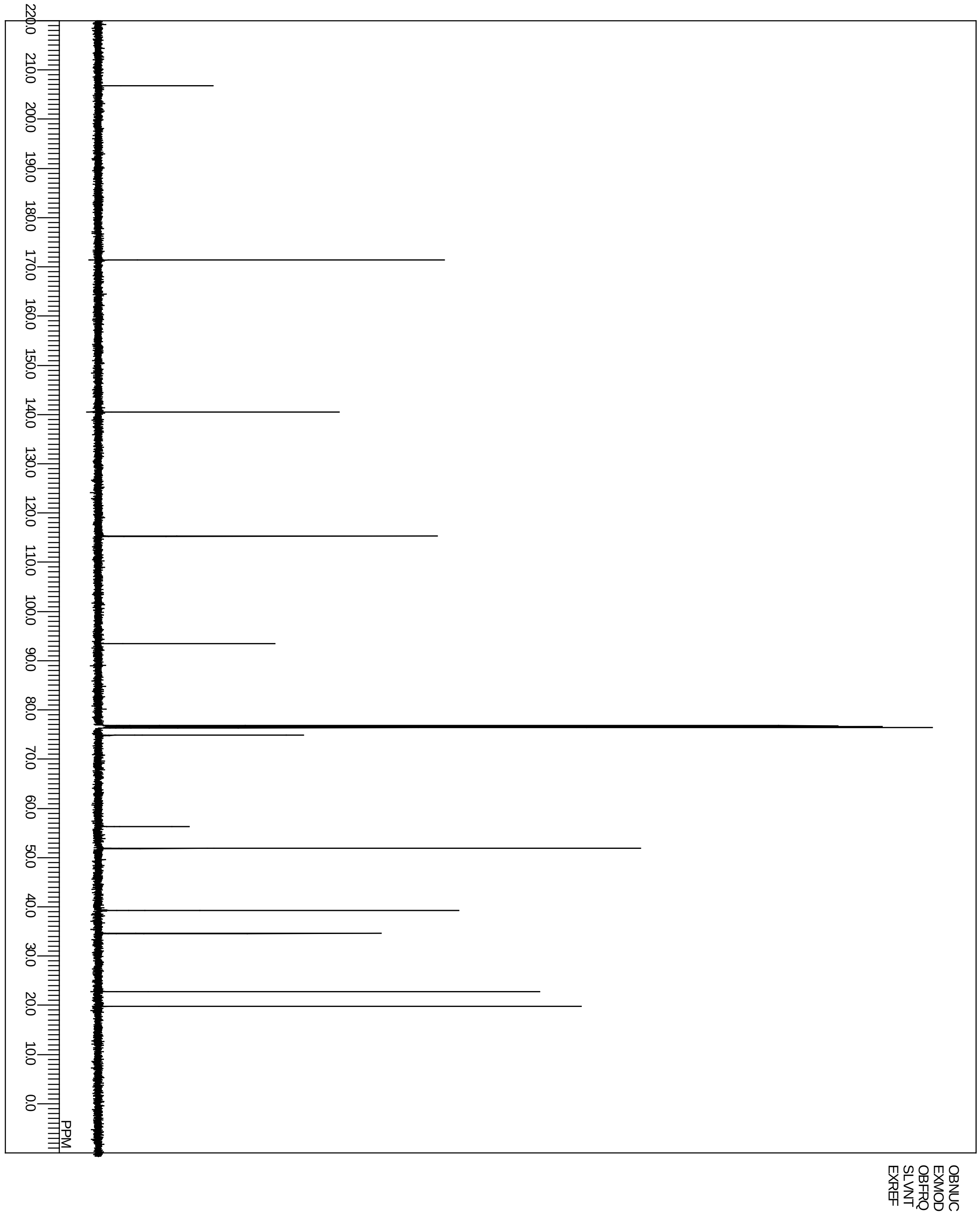
OBNUC 13C
EXMOD single pulse dec
OBFRQ 150.92 MHz
SLVNT CDCL3
EXREF 77.00 ppm
PPM

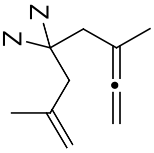
Z
Z

7a

Z = CO_2Me

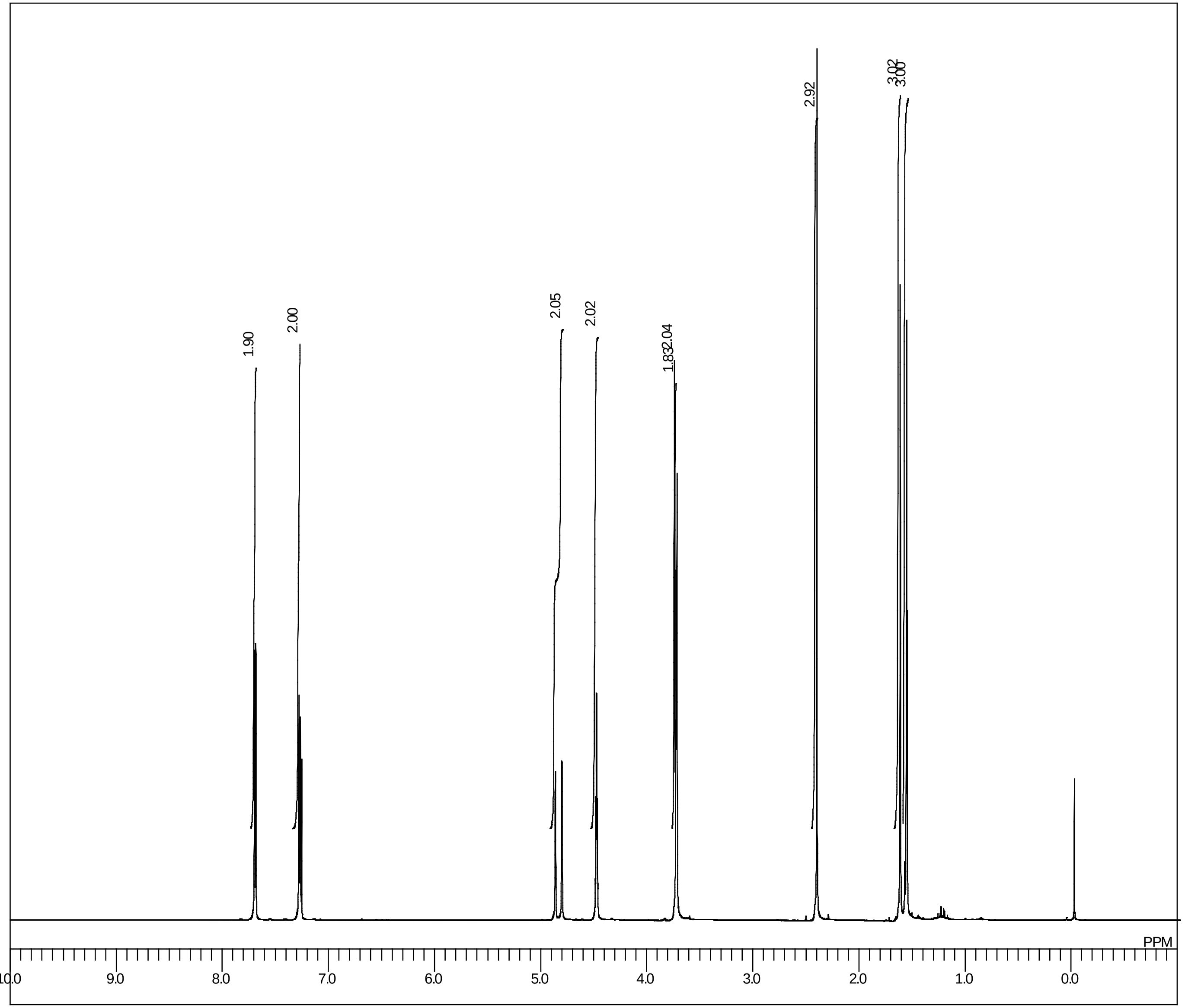

OBNUC 1H
EXMOD single_pulse.ex2
OBFRQ 600.17 MHz
SLVNT CDCL3
EXREF 7.26 ppm

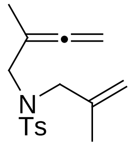

7b

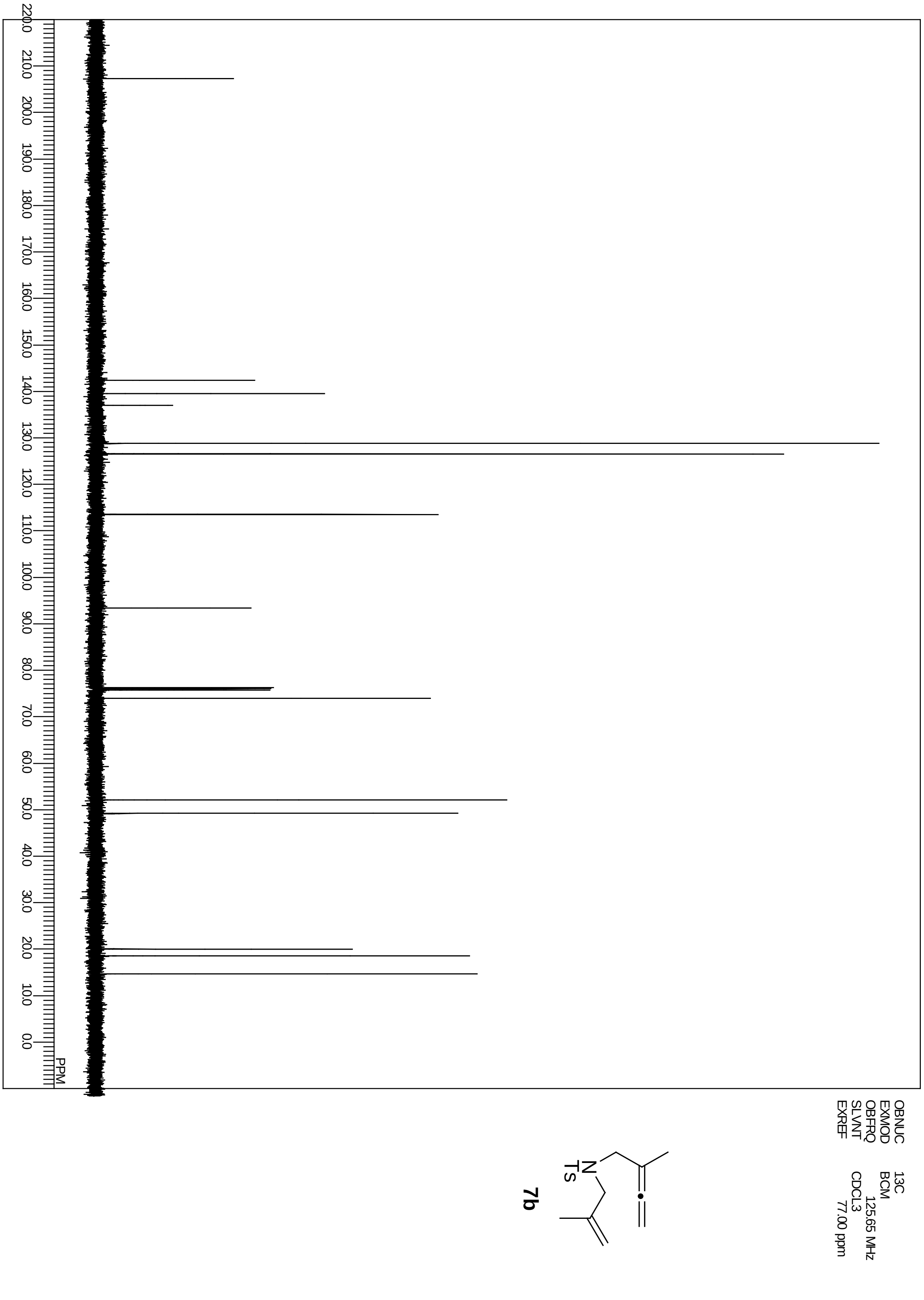
OBNUC 13C
EXMOD BCM
OBFRQ 125.65 MHz
SLVNT CDCL3
EXREF 77.00 ppm
PPM
N
Ts
7b

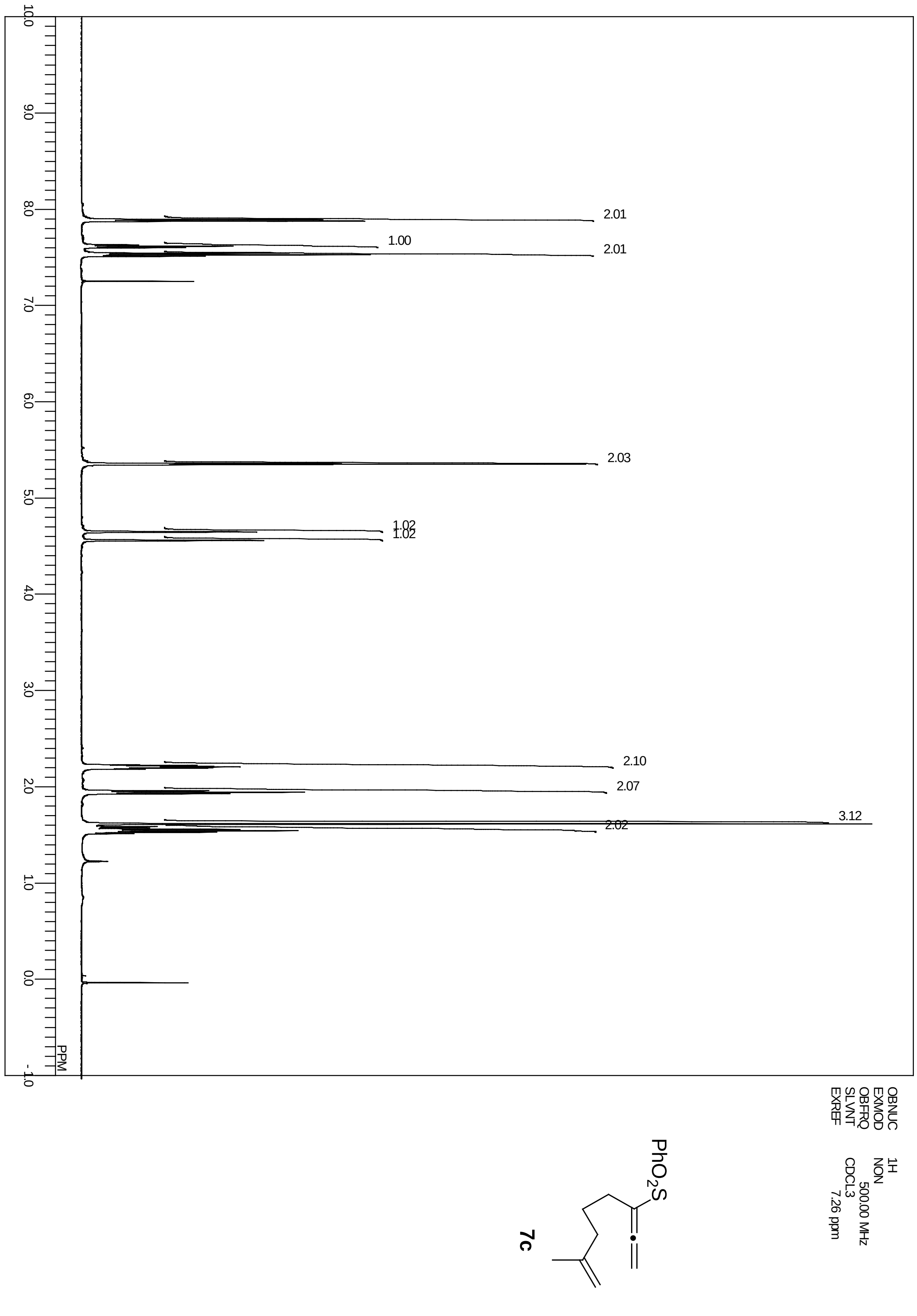

OBNUC 1H
EXMOD NON
OBFRQ 500.00 MHz
SLVNT CDCL3
EXREF 7.26 ppm
PPM
PhO2S
2.01
1.00
2.01
2.03
1.02
1.02
2.10
2.07
3.12
2.02

7c

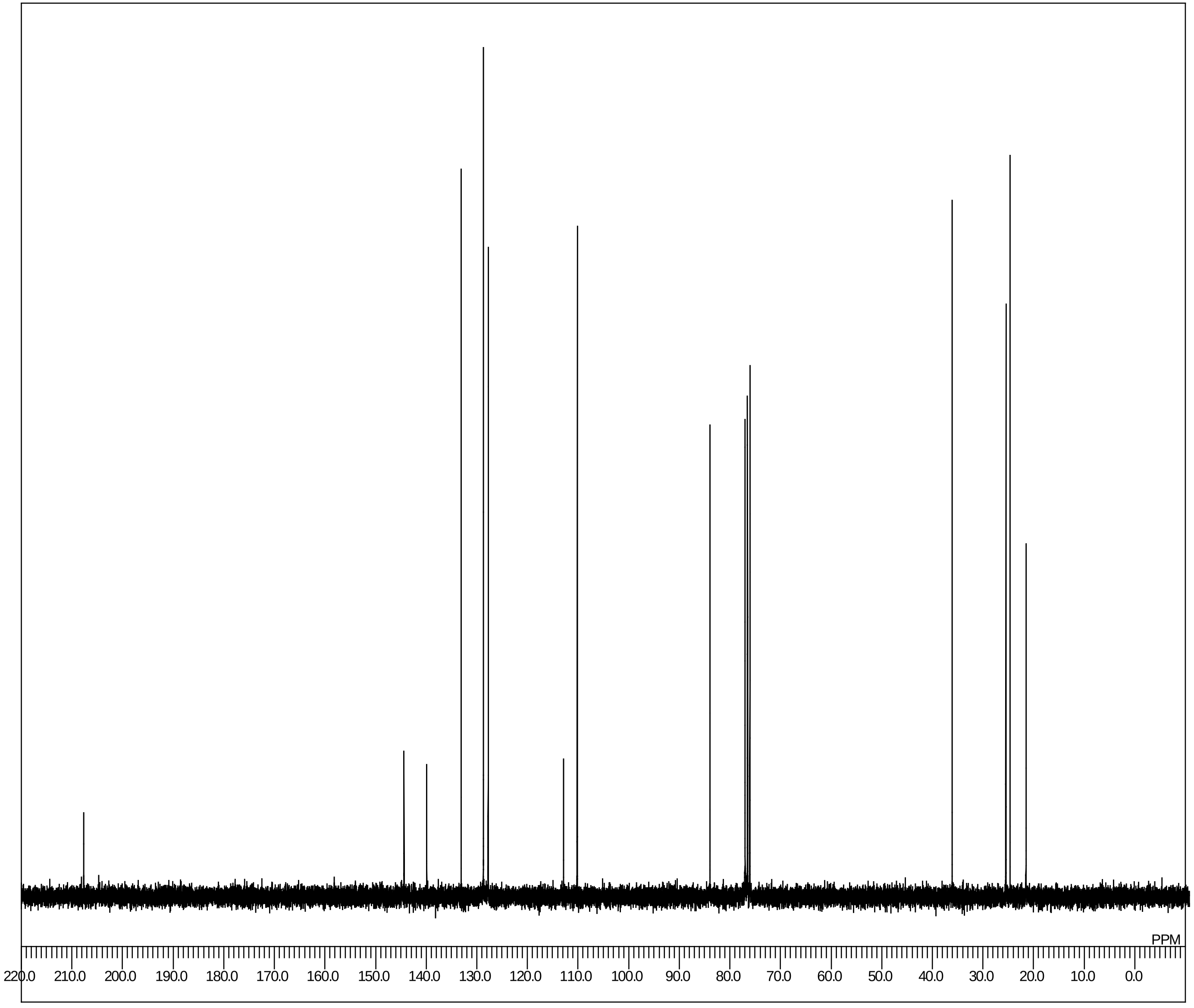

OBNUC	13C
EXMOD	BCM
OBFRQ	67.80 MHz
SLVNT	CDCL3
EXREF	77.00 ppm

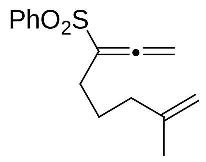

7c

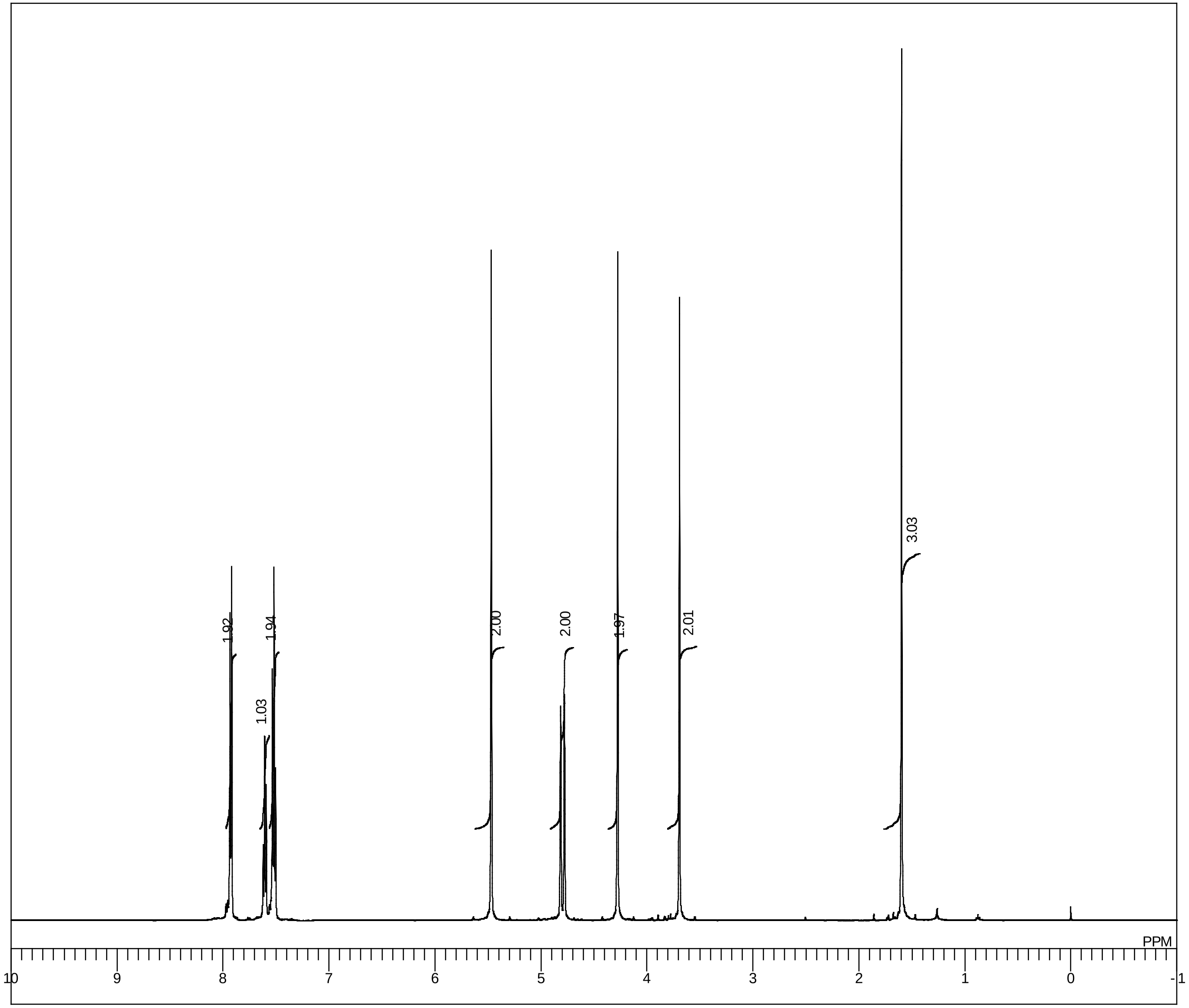
1.92
1.03
1.94
2.00
2.00
1.97
2.01
3.03
PPM
10
9
8
7
6
5
4
3
2
1
0
-1
OBNUC 1H
EXMOD NON
OBFRQ 500.00 MHz
SLVNT CDCL3
EXREF 0.00 ppm

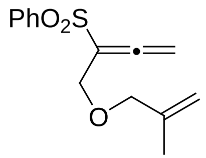
PhO2S
O

7d

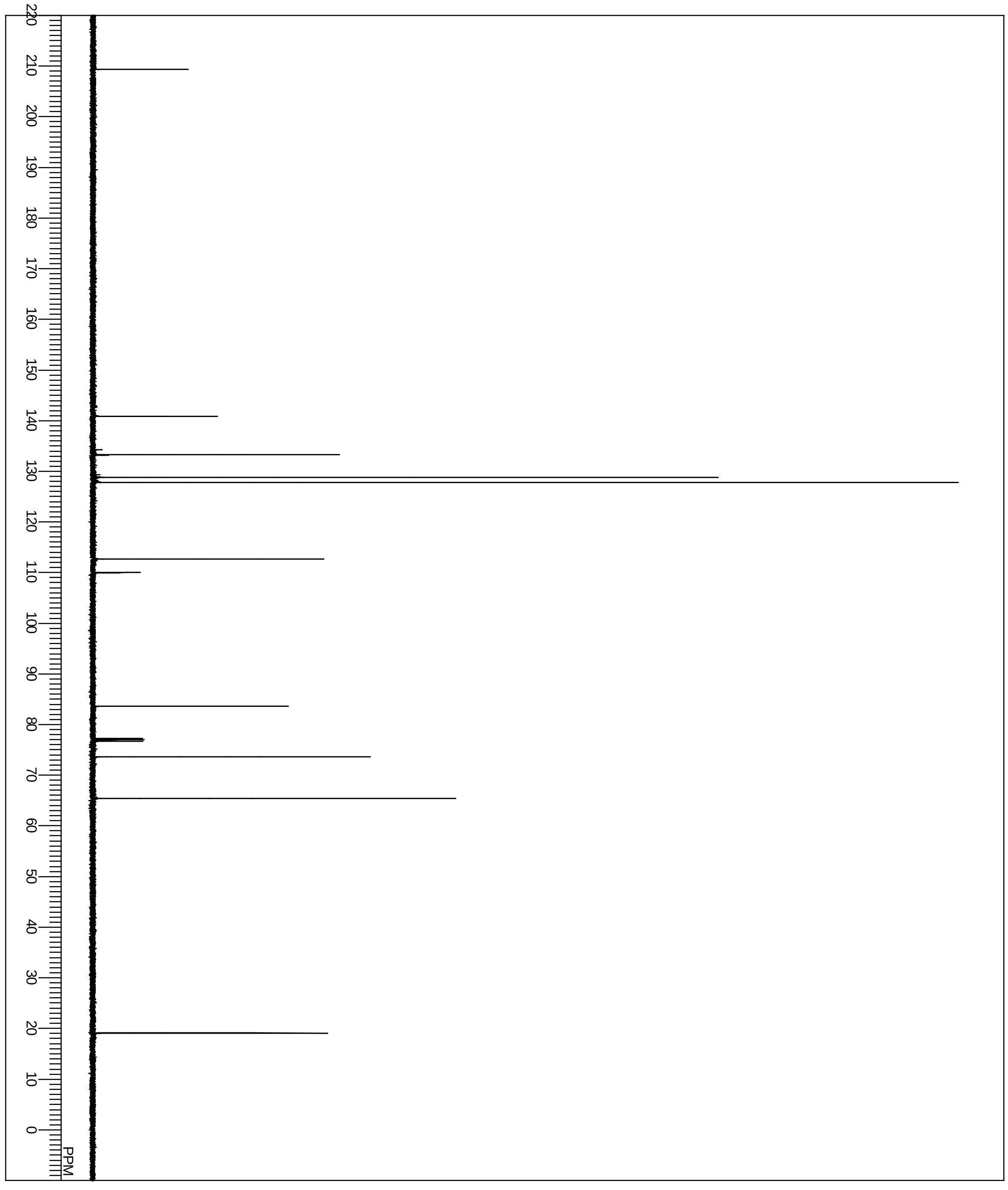

OBNUC 13C
EXMOD BCM
OBFRQ 125.65 MHz
SLVNT CDCL3
EXREF 77.00 ppm

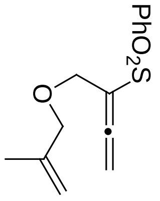

7d

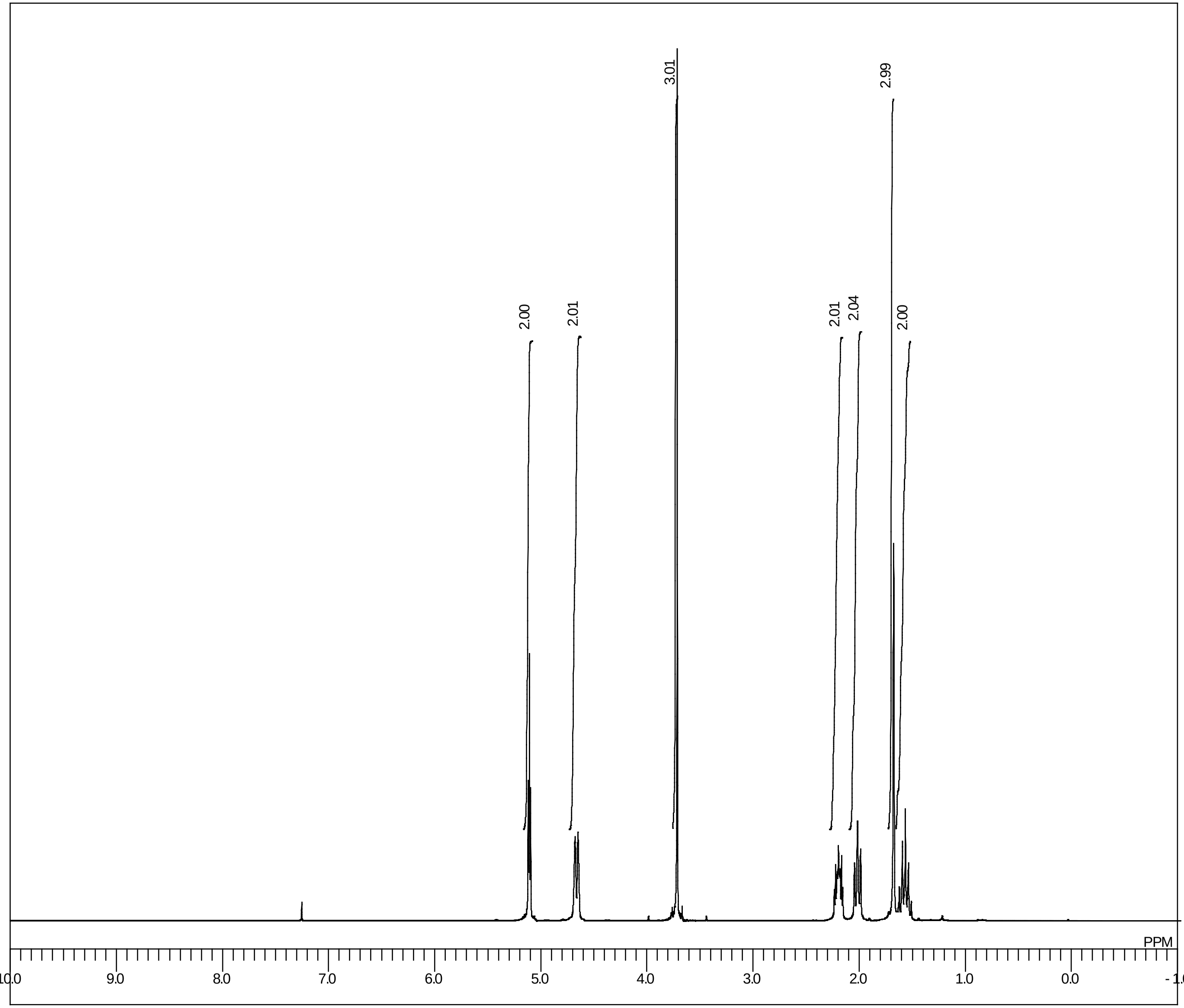

OBNUC	1H
EXMOD	NON
OBFRQ	270.05 MHz
SLVNT	CDCL3
EXREF	7.26 ppm

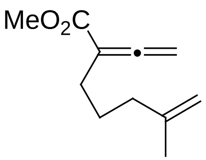

7e

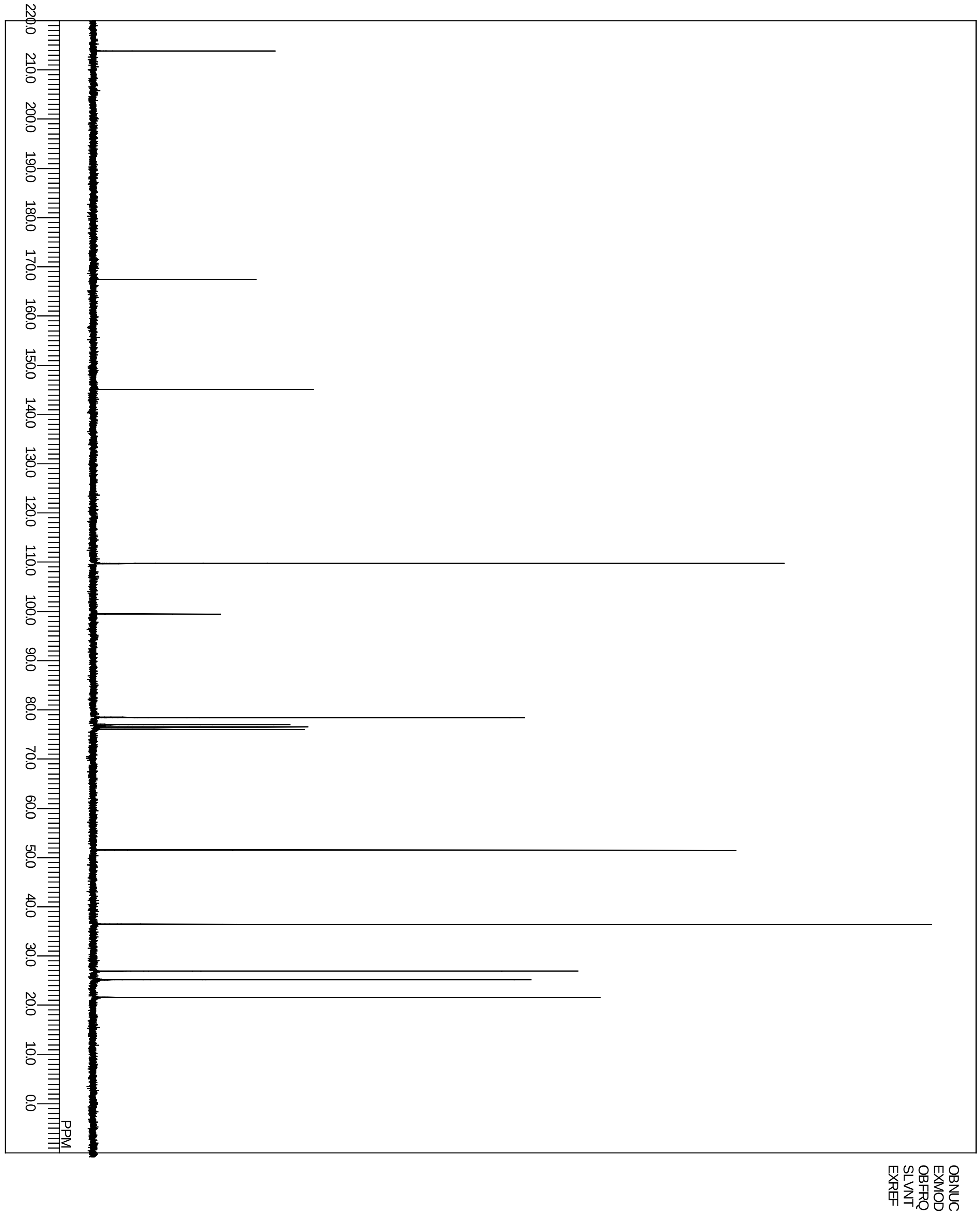

PPM
220.0
210.0
200.0
190.0
180.0
170.0
160.0
150.0
140.0
130.0
120.0
110.0
100.0
90.0
80.0
70.0
60.0
50.0
40.0
30.0
20.0
10.0
0.0
OBNUC 13C
EXMOD BCM
OBFRQ 67.80 MHz
SLVNT CDCL3
EXREF 77.00 ppm

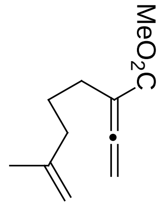

MeO2C

7e

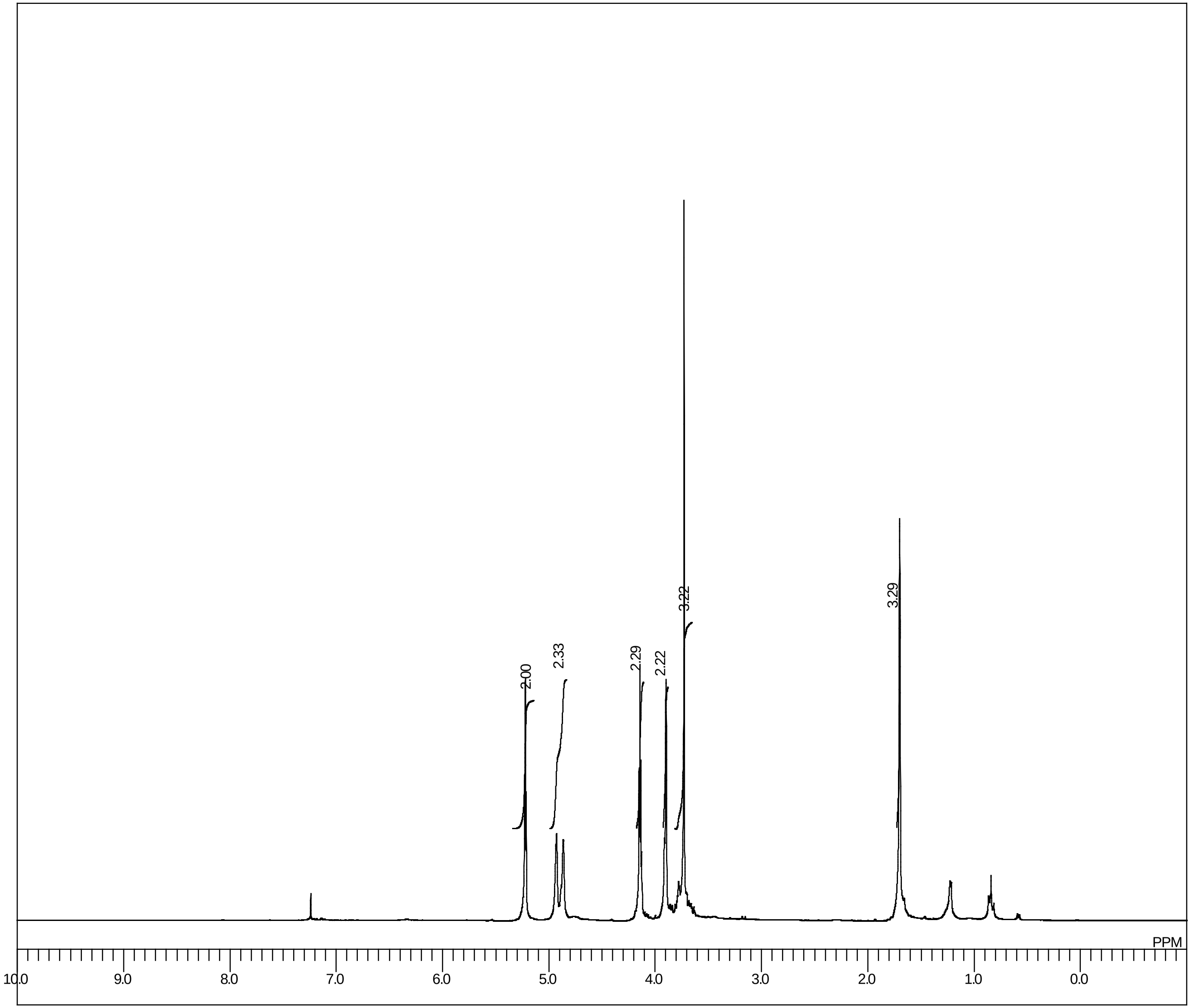

OBNUC 1H
EXMOD NON
OBFRQ 270.05 MHz
SLVNT CDCL3
EXREF 7.24 ppm

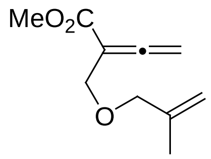

7f

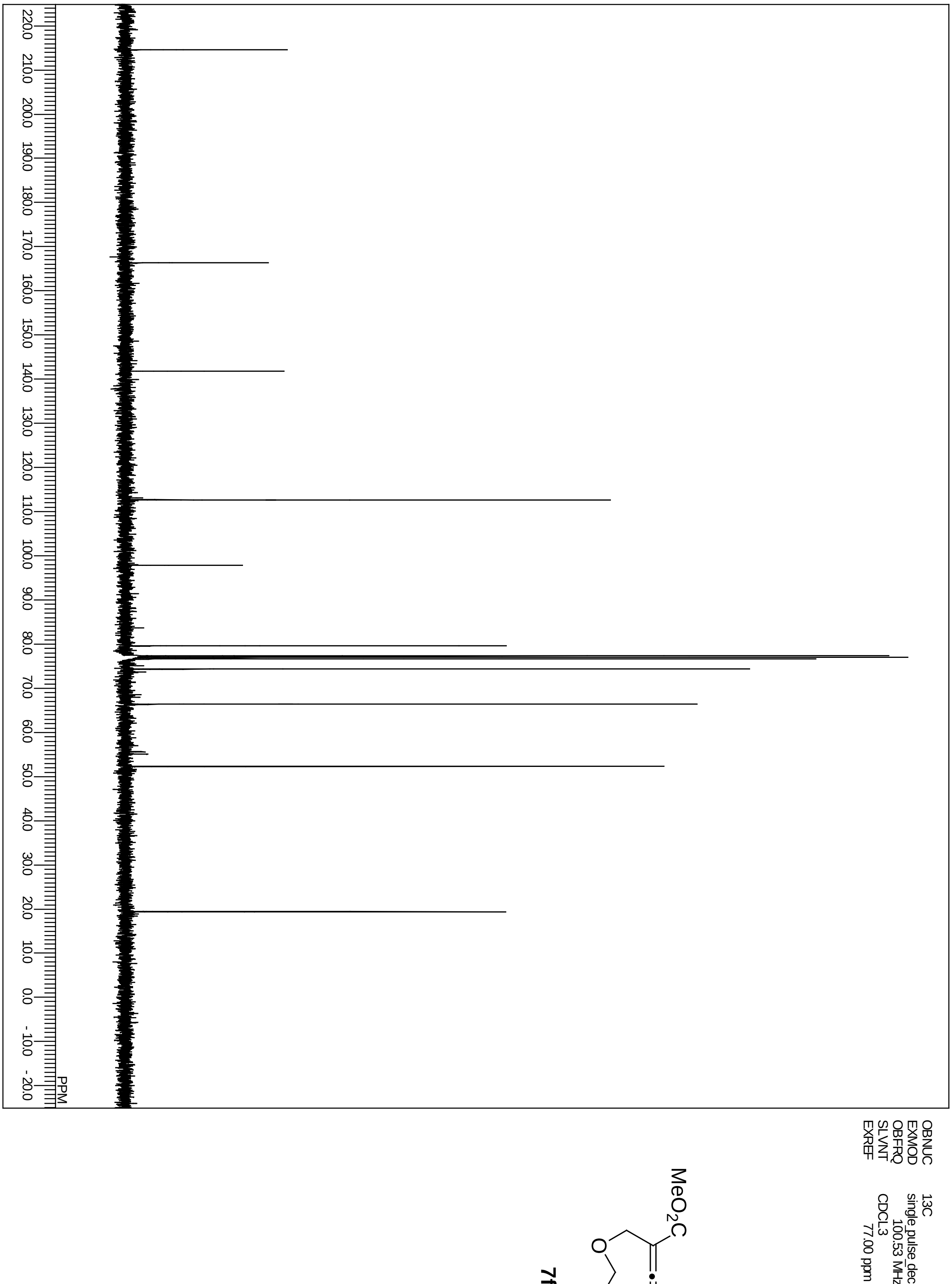
OBNUC 13C
EXMOD single pulse dec
OBFRQ 100.53 MHz
SLVNT CDCL3
EXREF 77.00 ppm
PPM

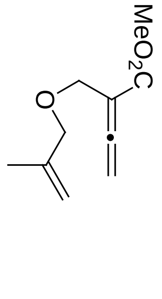
MeO2C

7f

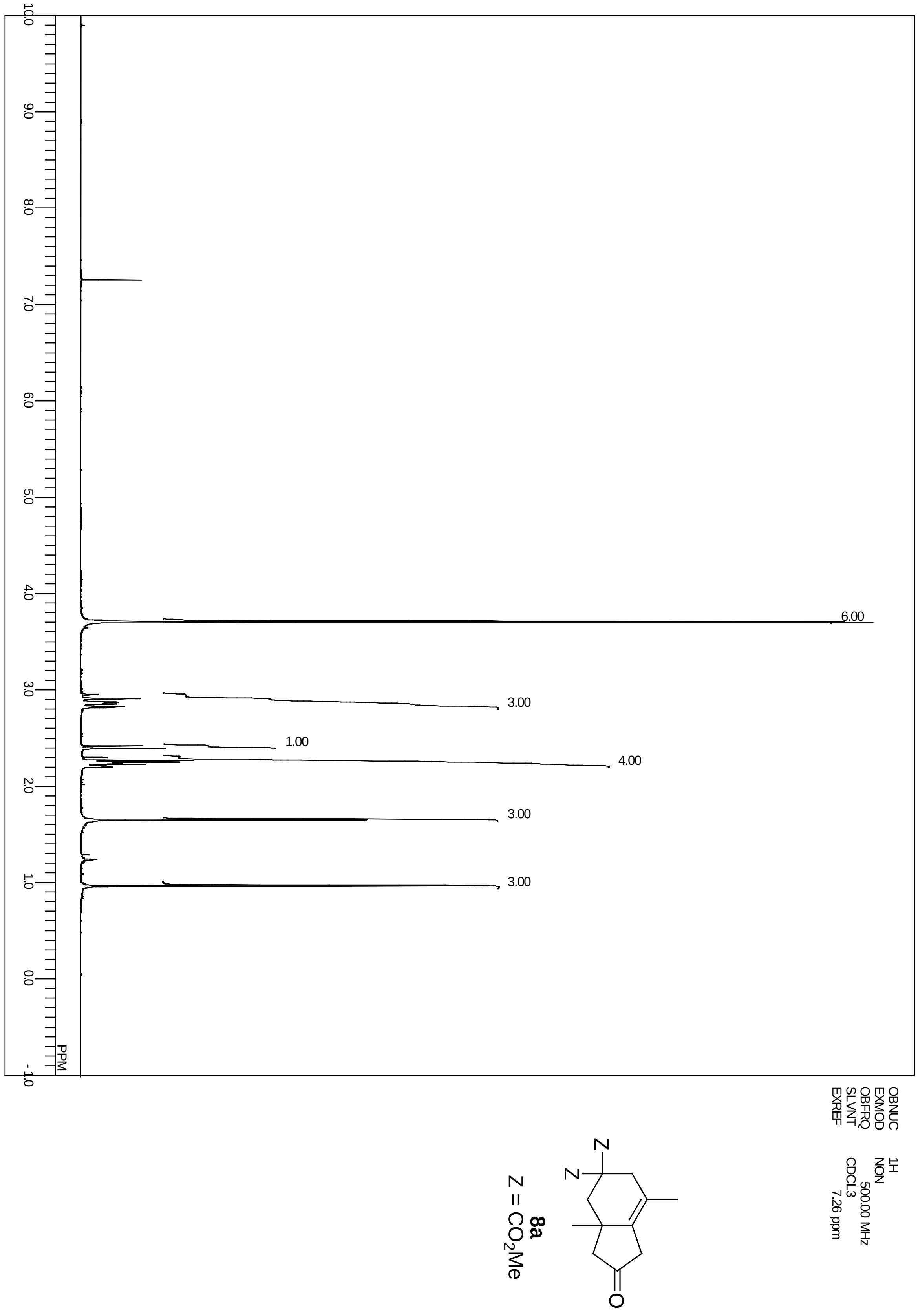
10.0
9.0
8.0
7.0
6.0
5.0
4.0
3.0
2.0
1.0
0.0
- 1.0
PPM
6.00
3.00
1.00
4.00
3.00
3.00
OBNUC 1H
EXMOD NON
OBFRQ 500.00 MHz
SLVNT CDCL3
EXREF 7.26 ppm
Z
Z
O
8a
Z = CO2Me

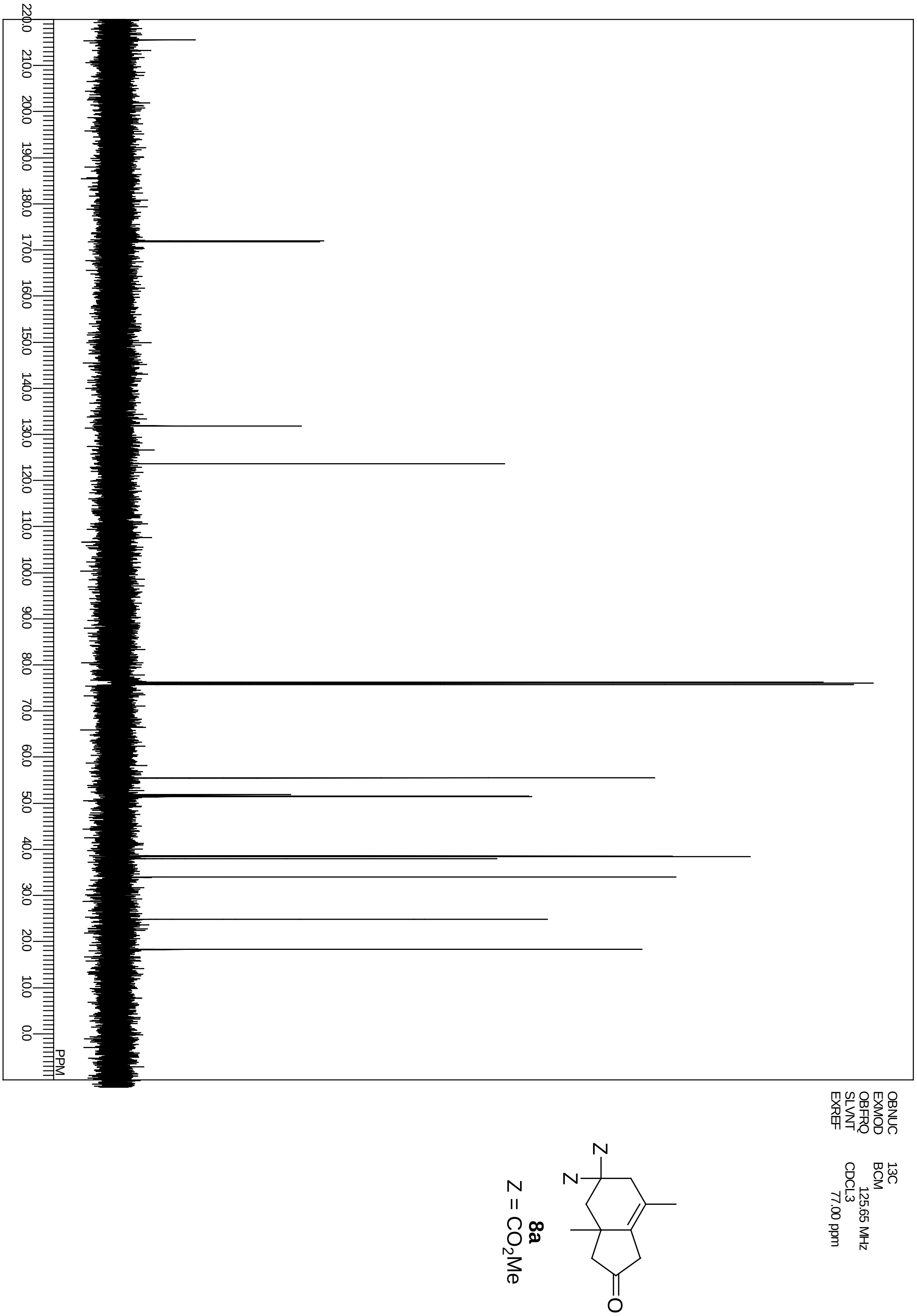
OBNUC 13C
EXMOD BCM
OBFRQ 125.65 MHz
SLVNT CDCL3
EXREF 77.00 ppm
PPM
Z
Z
O
8a
Z = CO2Me

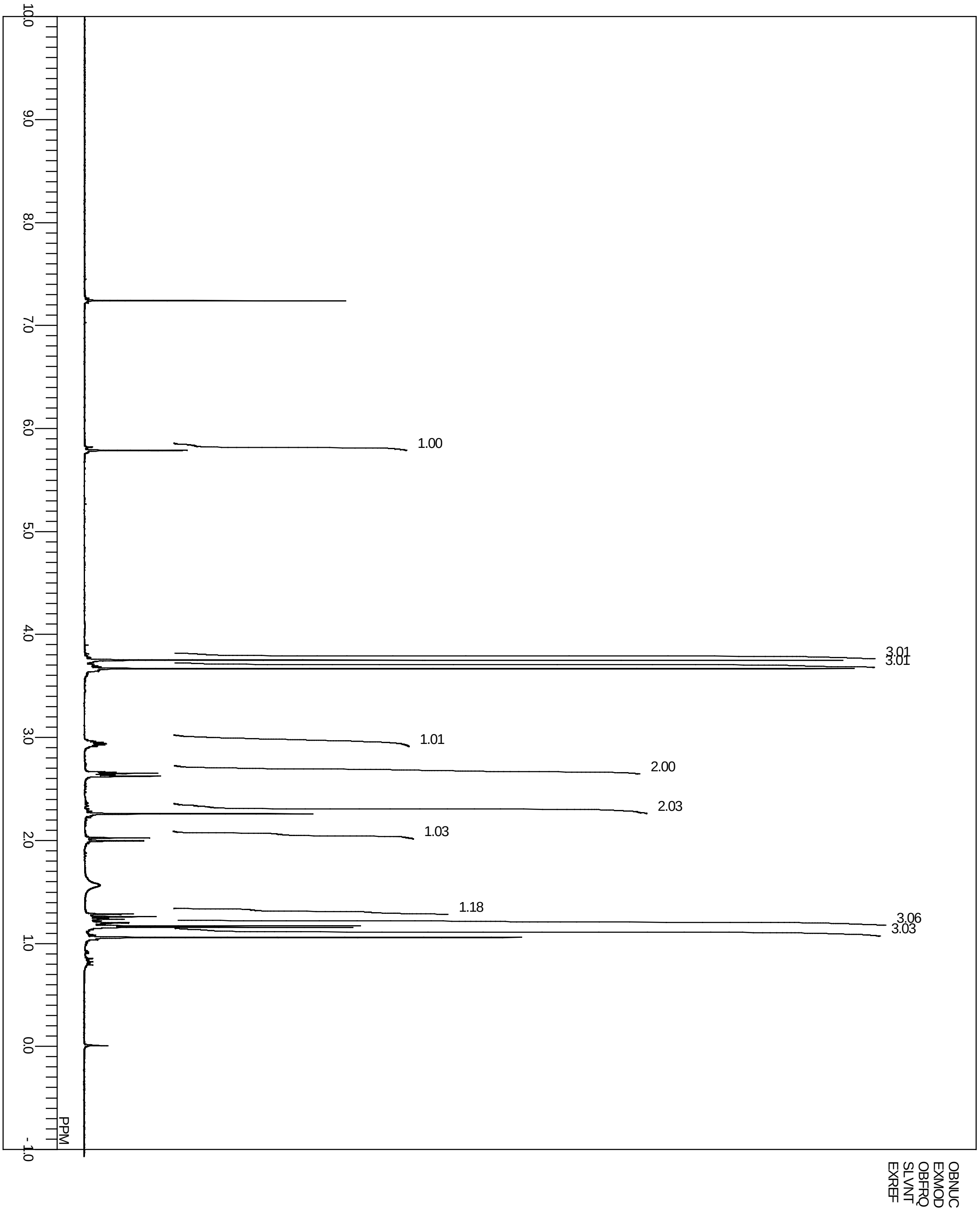
1.00
3.01
3.01
1.01
2.00
2.03
1.03
1.18
3.06
3.03
PPM
OBNUC 1H
EXMOD NON
OBFRQ 500.00 MHz
SLVNT CDCL3
EXREF 7.26 ppm

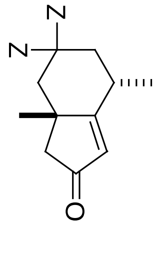
Z
Z
O

9a

Z = CO_2Me

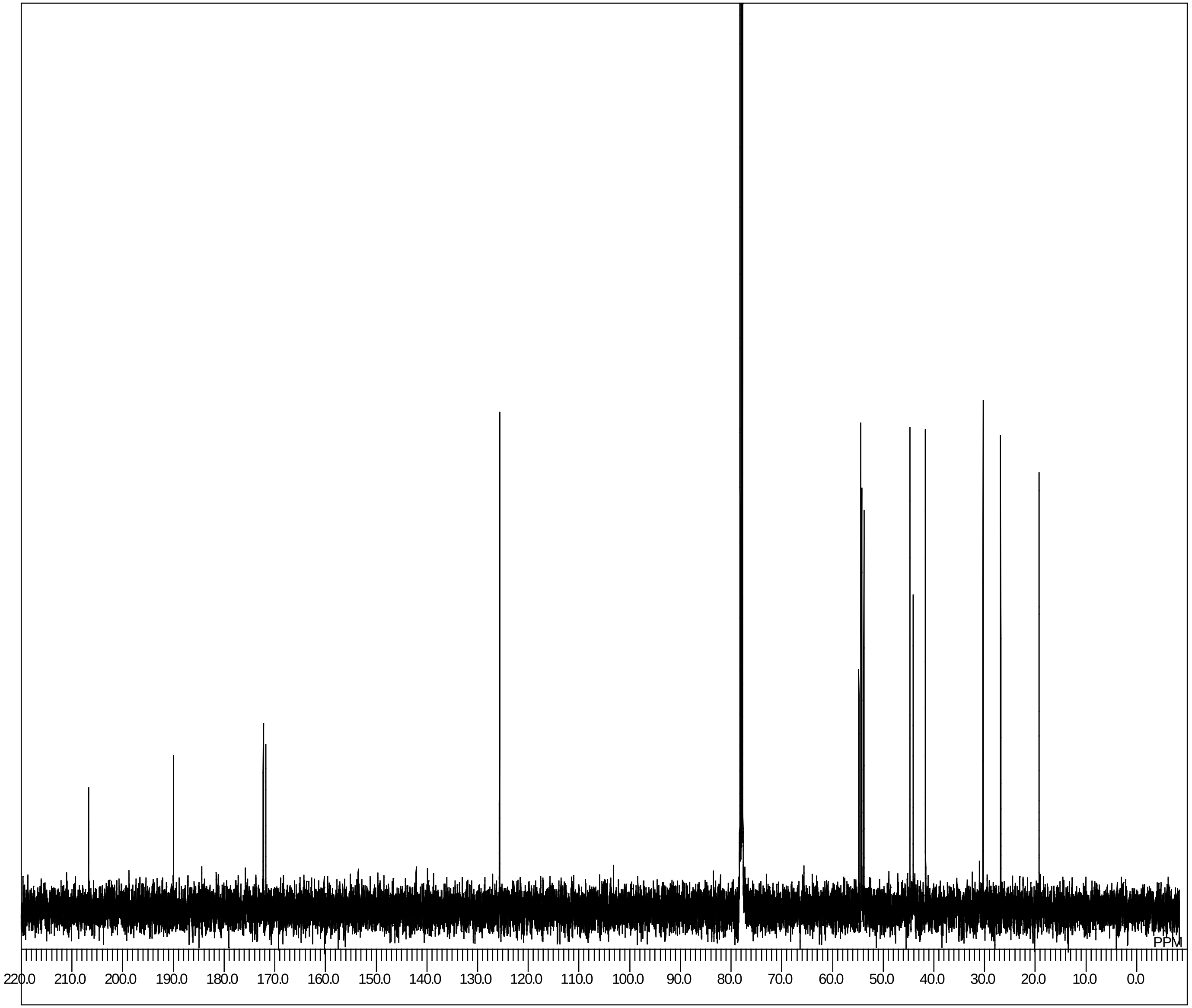

OBNUC	13C
EXMOD	BCM
OBFRQ	125.65 MHz
SLVNT	CDCL3
EXREF	77.00 ppm

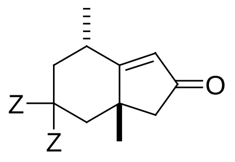

9a
Z = CO_2Me

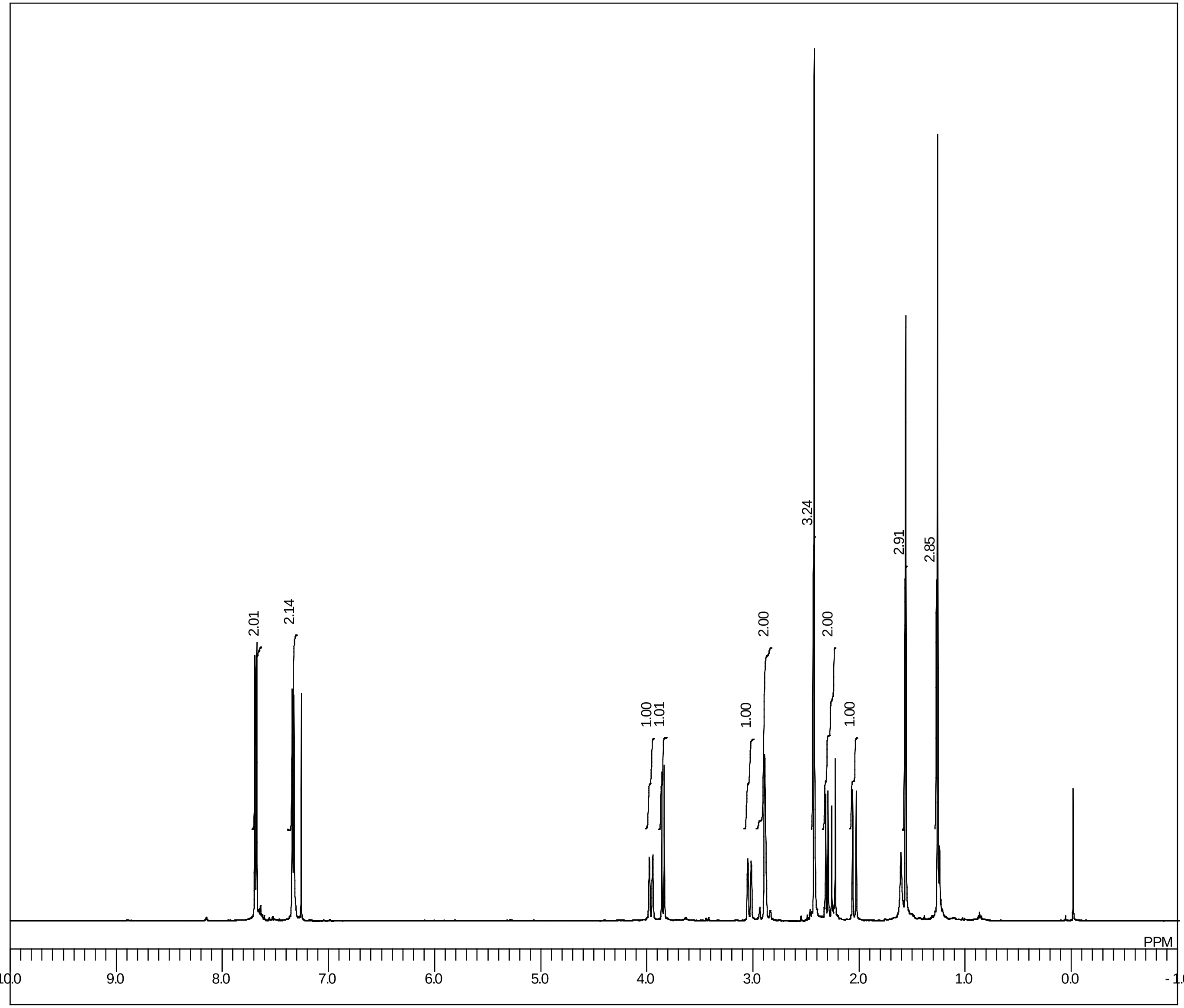

OBNUC 1H
EXMOD NON
OBFRQ 500.00 MHz
SLVNT CDCL3
EXREF 7.26 ppm

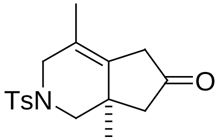

8b

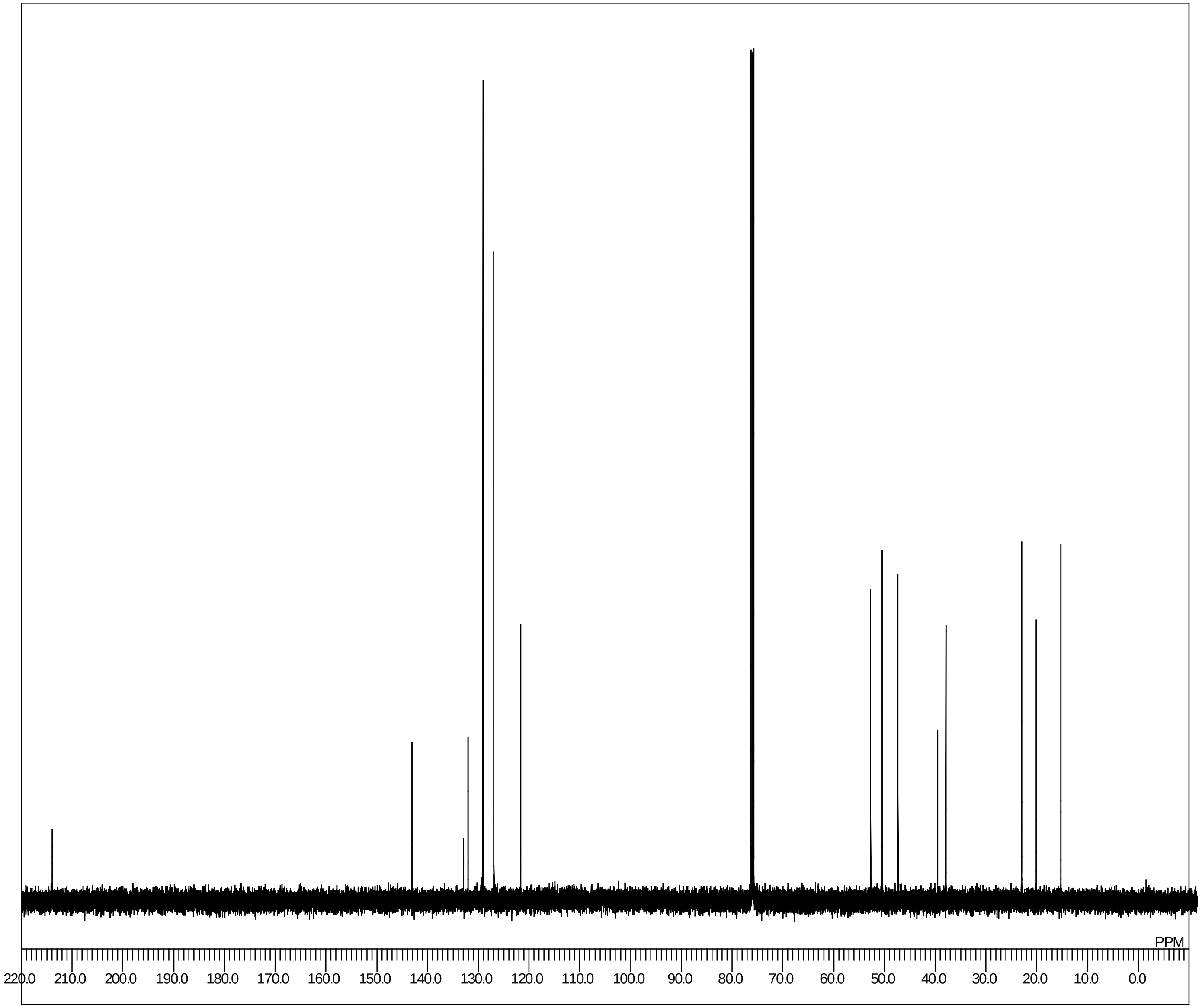

OBNUC 13C
EXMOD BCM
OBFRQ 125.65 MHz
SLVNT CDCL3
EXREF 77.00 ppm

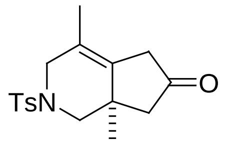

8b

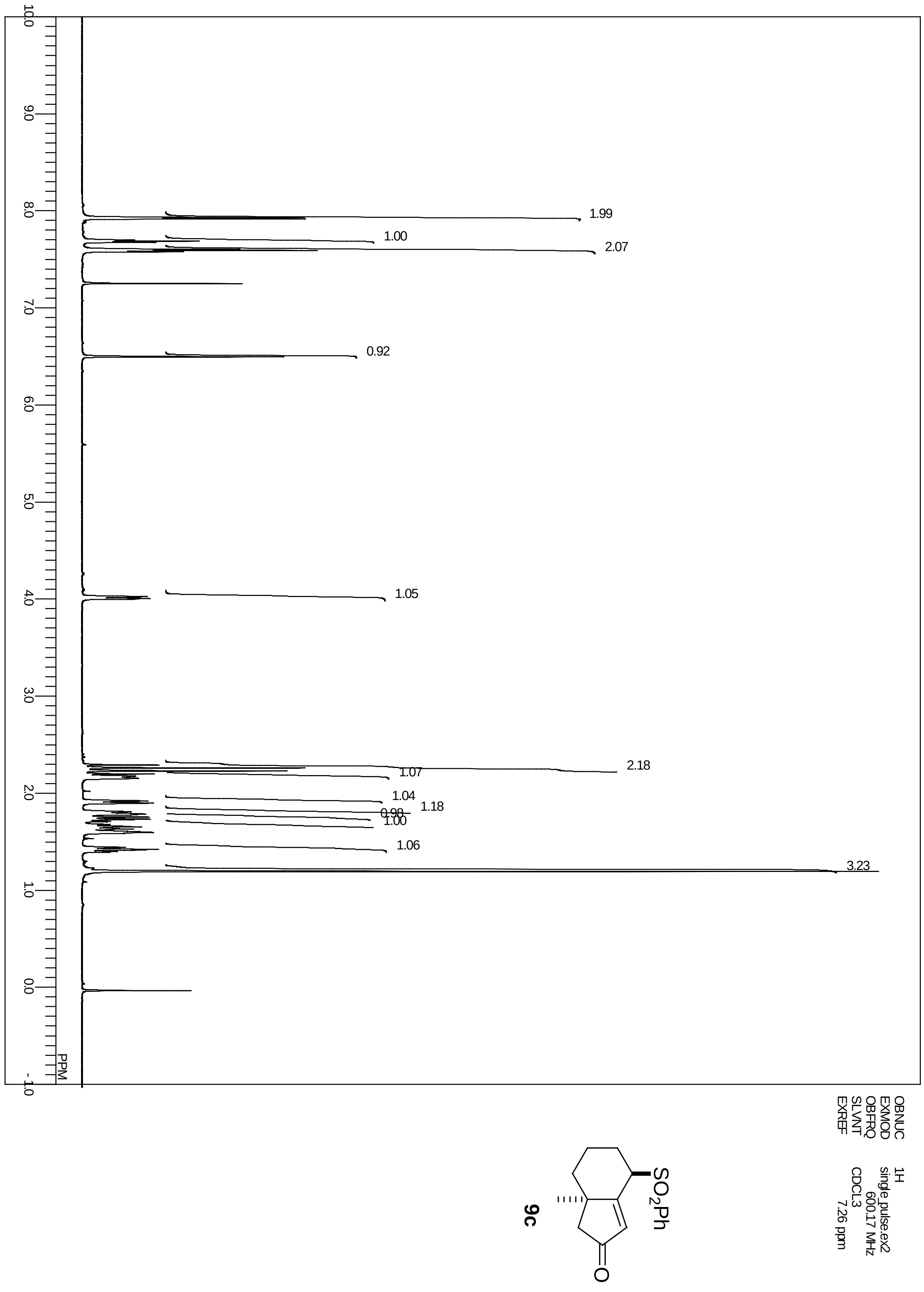
10.0
9.0
8.0
7.0
6.0
5.0
4.0
3.0
2.0
1.0
0.0
- 1.0
PPM
1.99
1.00
2.07
0.92
1.05
2.18
1.07
1.04
1.18
0.98
1.00
1.06
3.23
OBNUC 1H
EXMOD single_pulse.ex2
OBFRQ 600.17 MHz
SLVNT CDCL3
EXREF 7.26 ppm
SO2Ph
O
9c

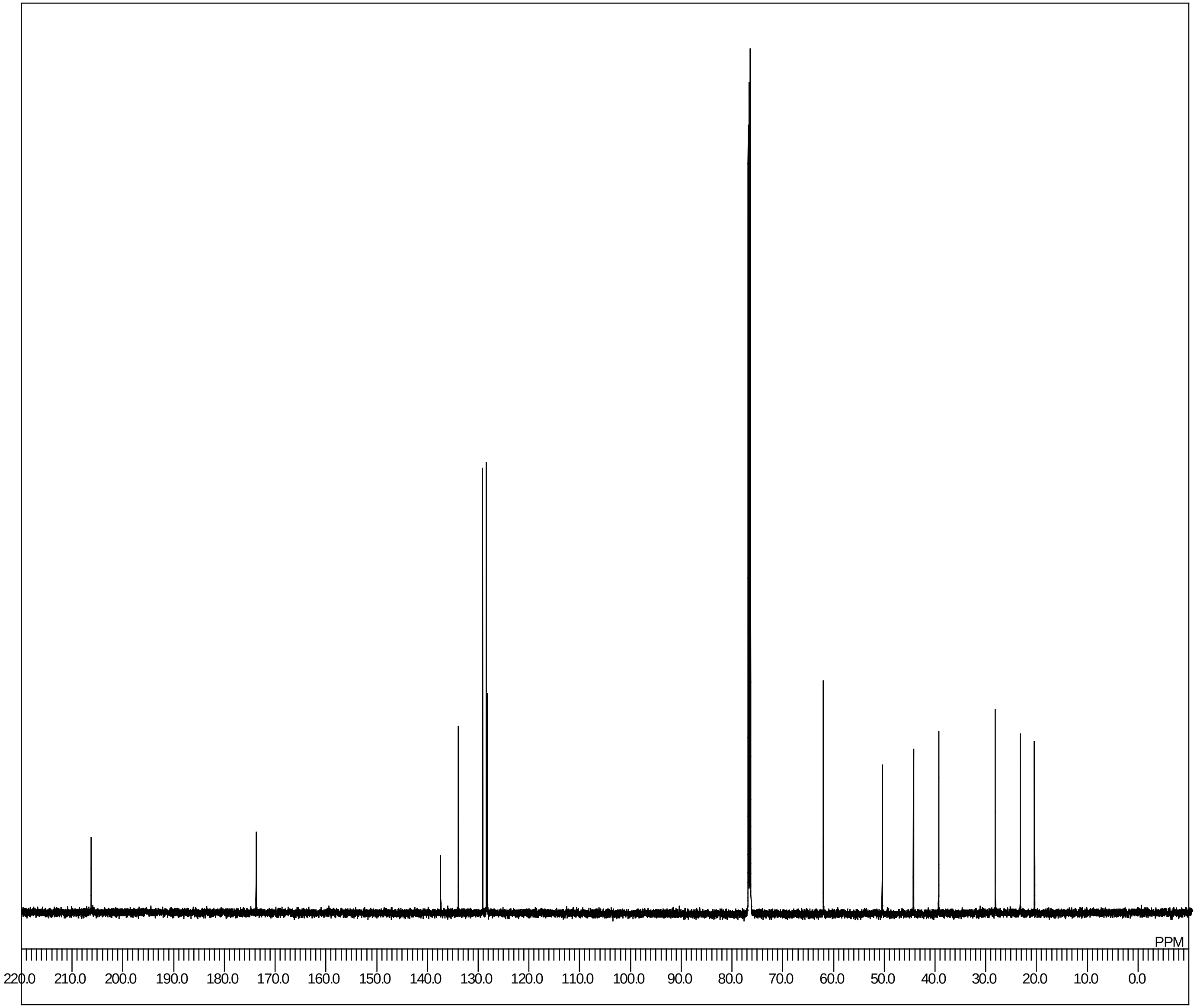

OBNUC	13C
EXMOD	single_pulse_dec
OBFRQ	150.92 MHz
SLVNT	CDCL3
EXREF	77.00 ppm

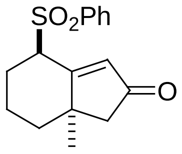

9c

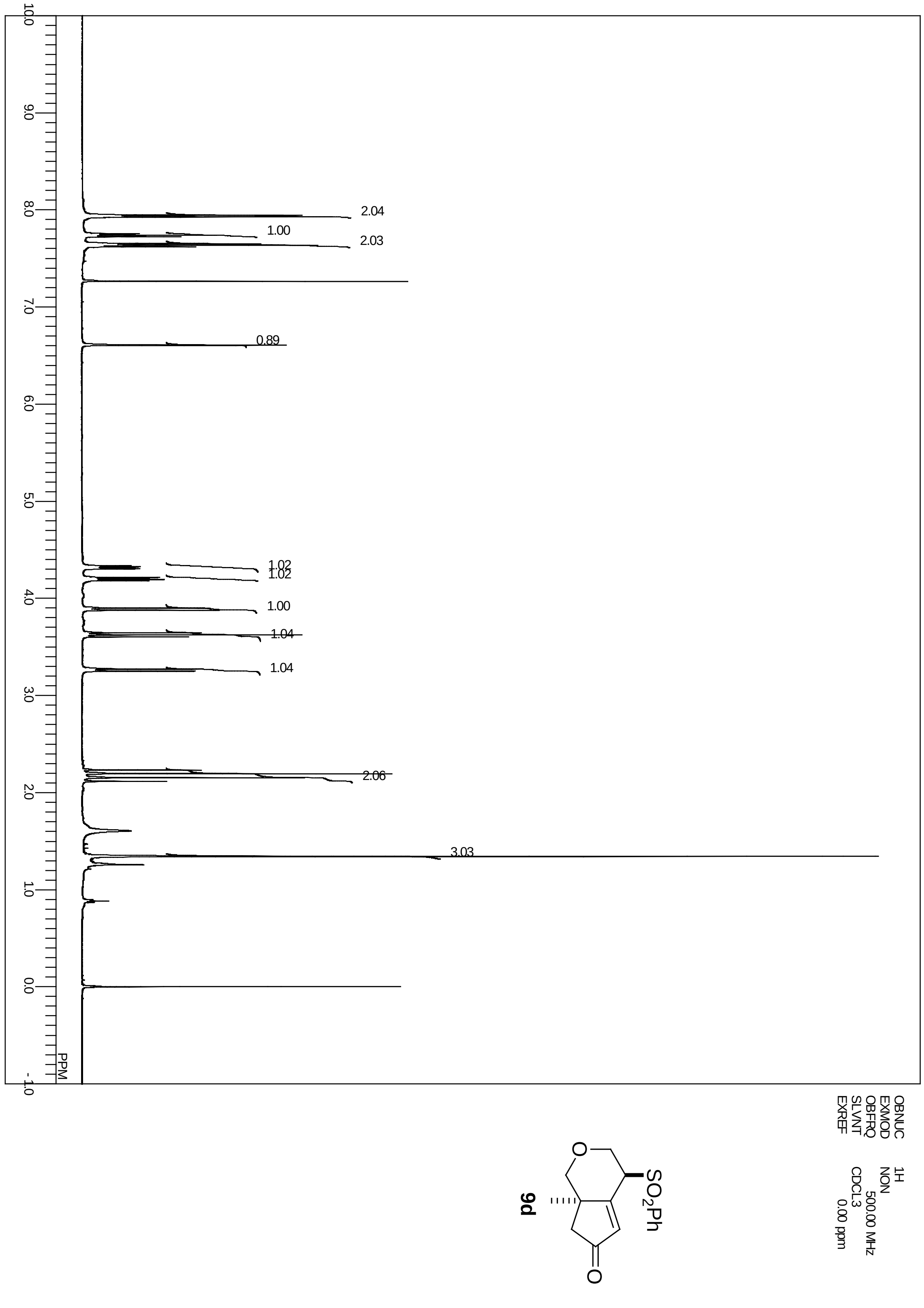
10.0
9.0
8.0
7.0
6.0
5.0
4.0
3.0
2.0
1.0
0.0
-1.0
PPM
2.04
1.00
2.03
0.89
1.02
1.02
1.00
1.04
1.04
2.06
3.03
OBNUC 1H
EXMOD NON
OBFRQ 500.00 MHz
SLVNT CDCL3
EXREF 0.00 ppm
SO2Ph
O
O
9d

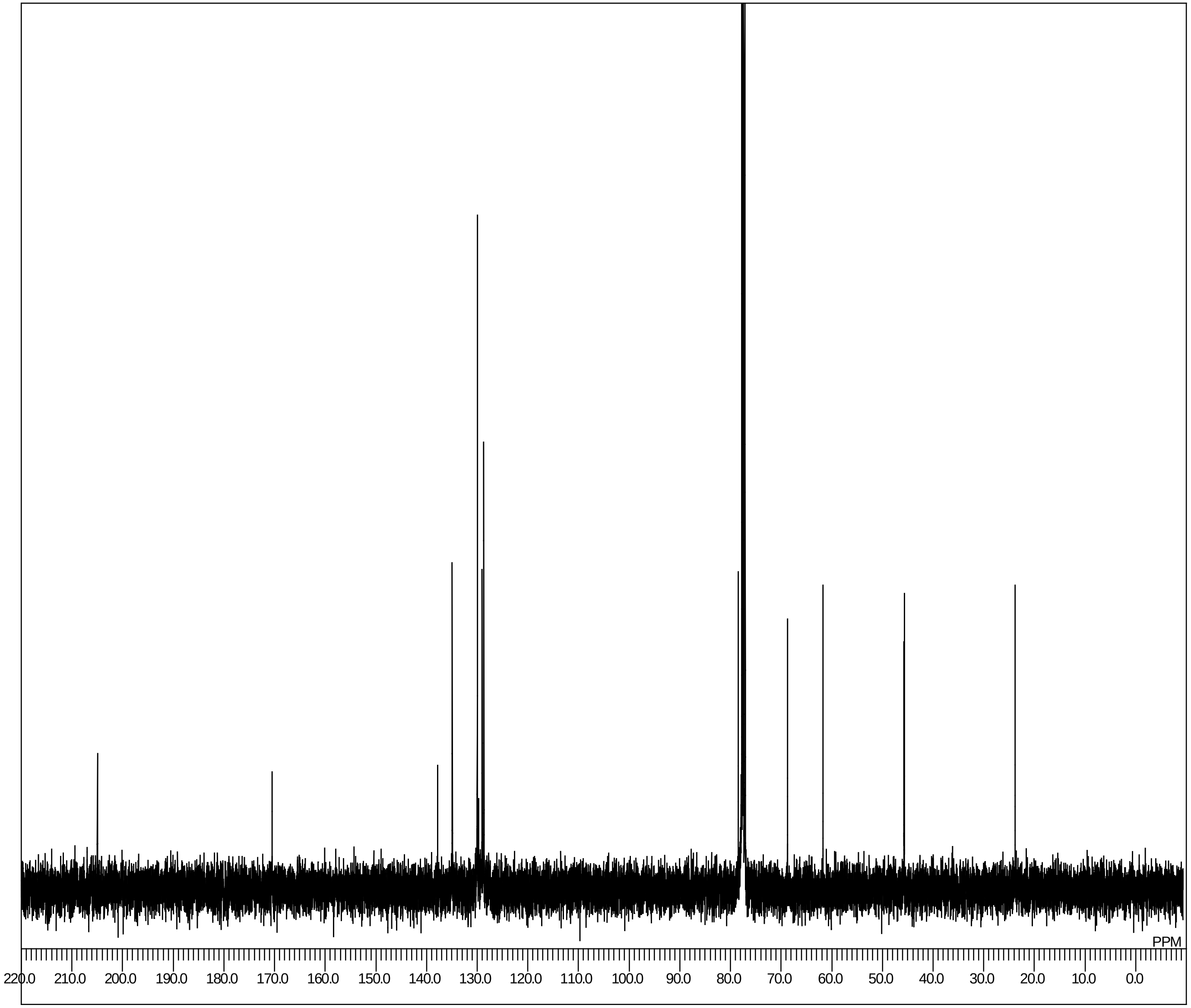

OBNUC 13C
EXMOD single_pulse_dec
OBFRQ 100.53 MHz
SLVNT CDCL3
EXREF 77.00 ppm

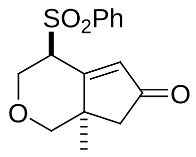

9d

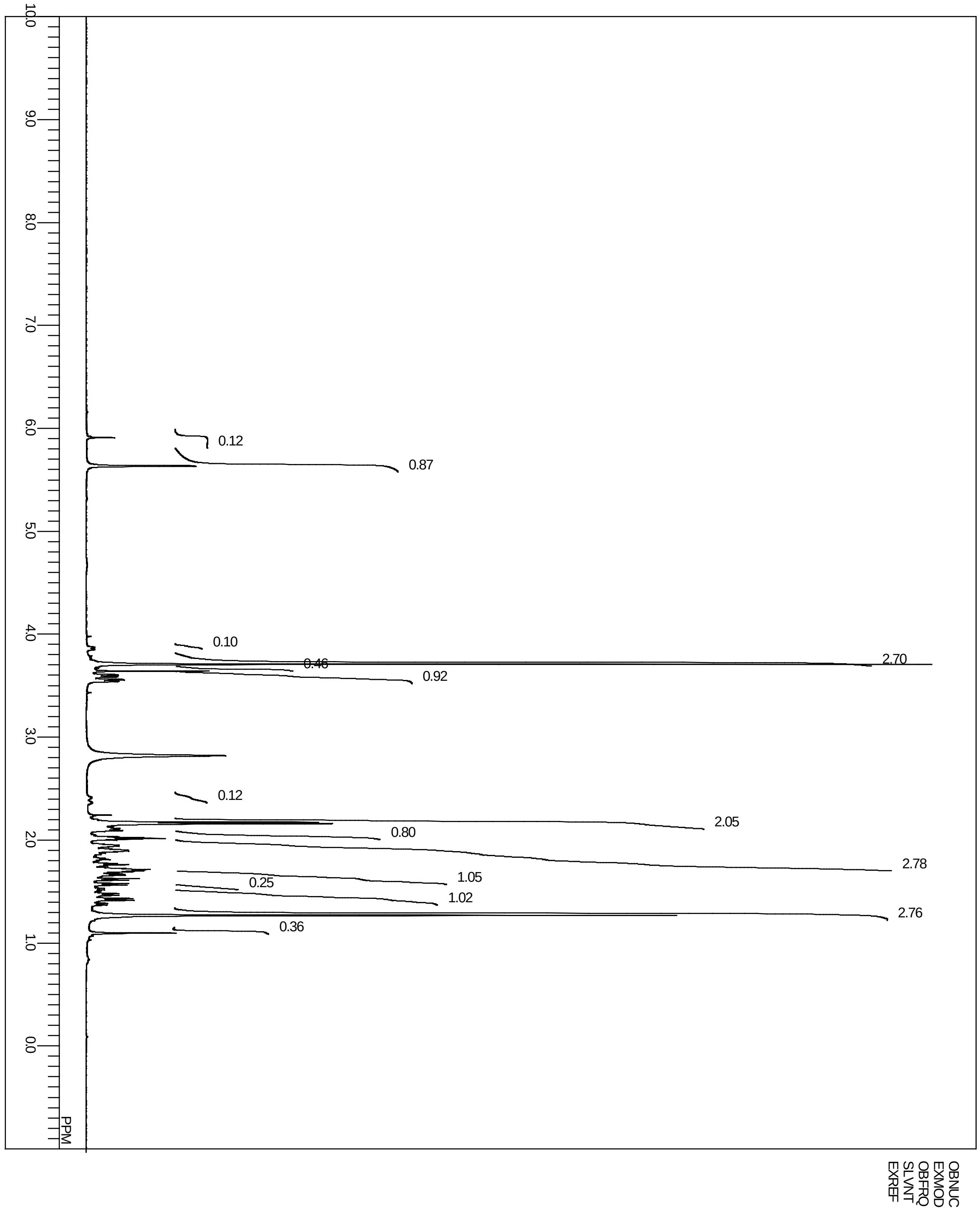
10.0
9.0
8.0
7.0
6.0
5.0
4.0
3.0
2.0
1.0
0.0
PPM
0.12
0.87
0.10
0.46
2.70
0.92
0.12
2.05
0.80
2.78
0.25
1.05
1.02
2.76
0.36
OBNUC 1H
EXMOD NON
OBFRQ 270.05 MHz
SLVNT ACETN
EXREF 2.84 ppm

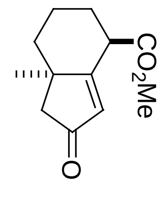
CO2Me
O

9e

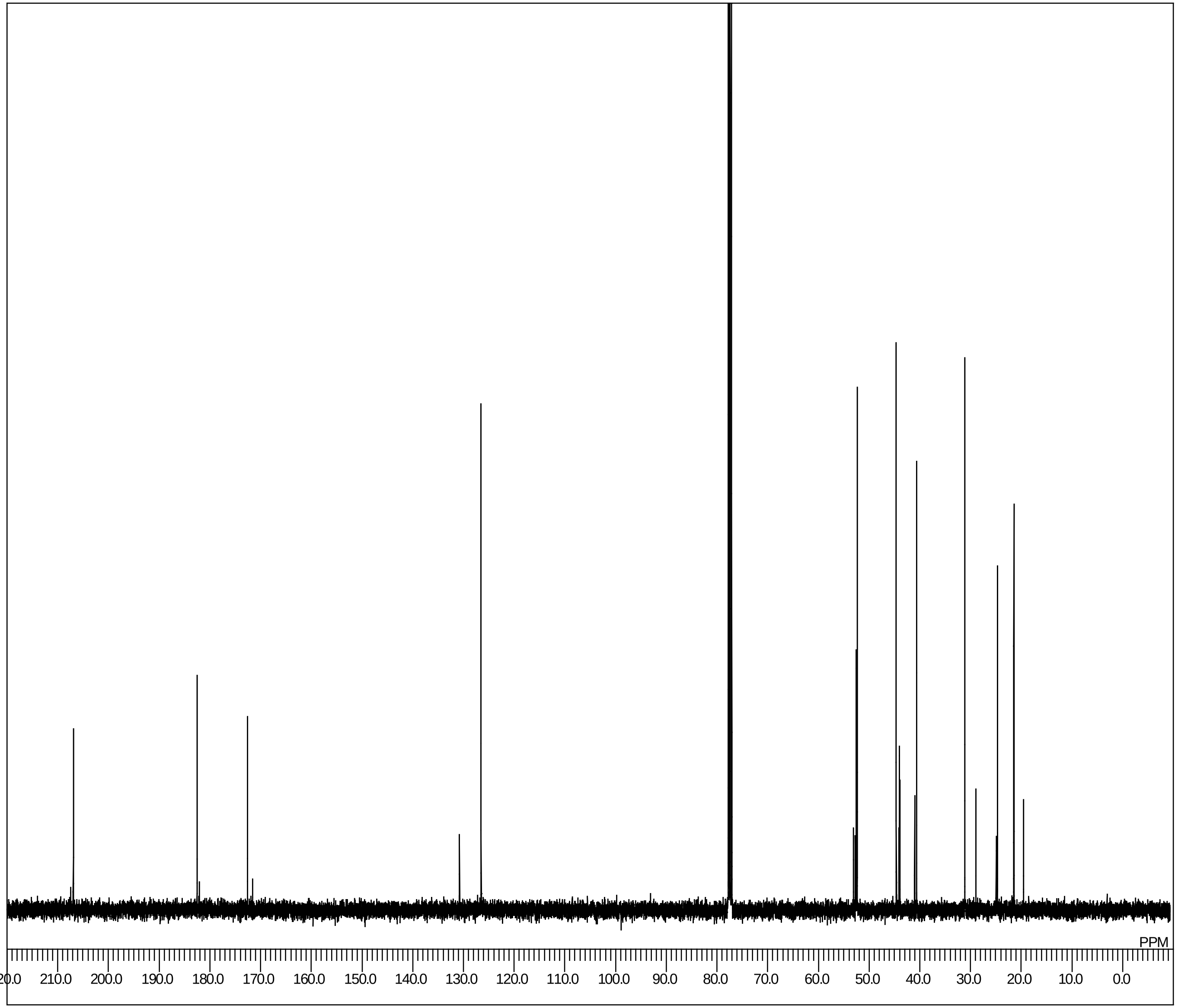

OBNUC 13C
EXMOD single_pulse_dec
OBFRQ 100.53 MHz
SLVNT CDCL3
EXREF 77.00 ppm

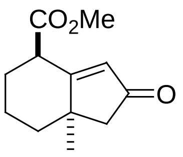

9e

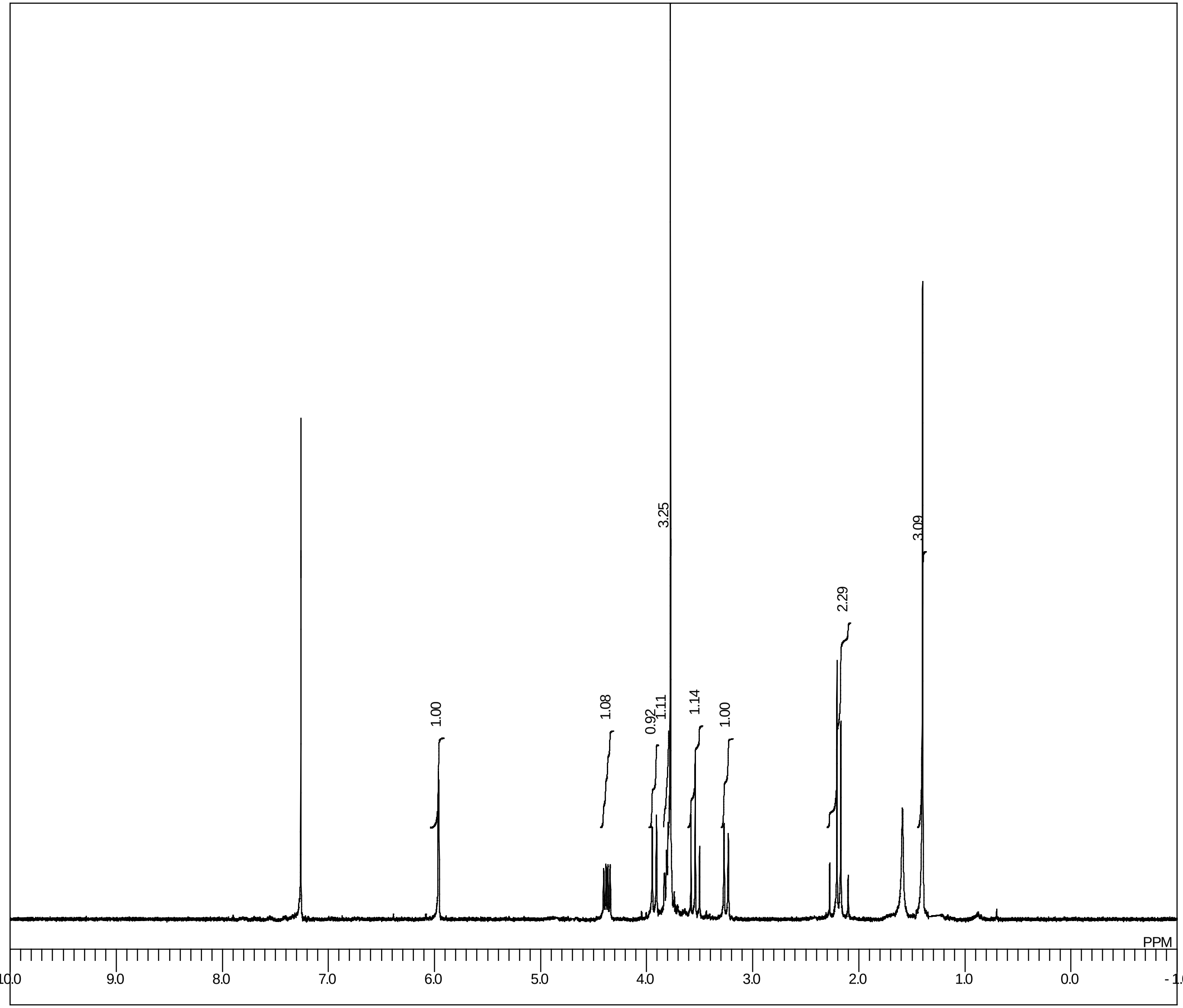

OBNUC 1H
EXMOD NON
OBFRQ 270.05 MHz
SLVNT CDCL3
EXREF 7.26 ppm

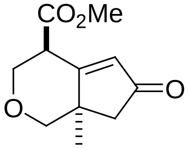

9f

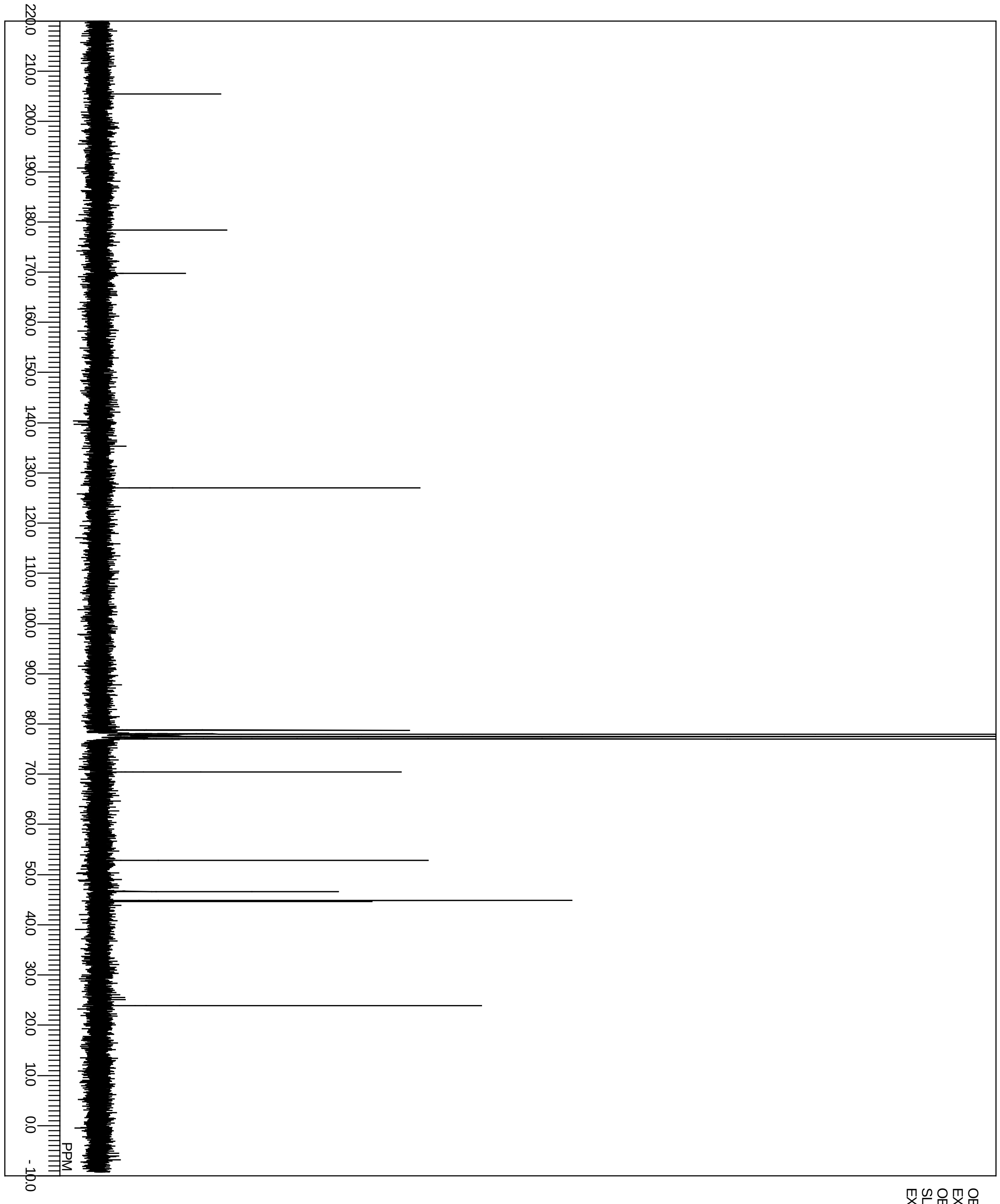

OBNUC 13C
EXMOD BCM
OBFRQ 67.80 MHz
SLVNT CDCL3
EXREF 77.00 ppm

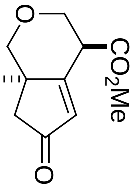

9f

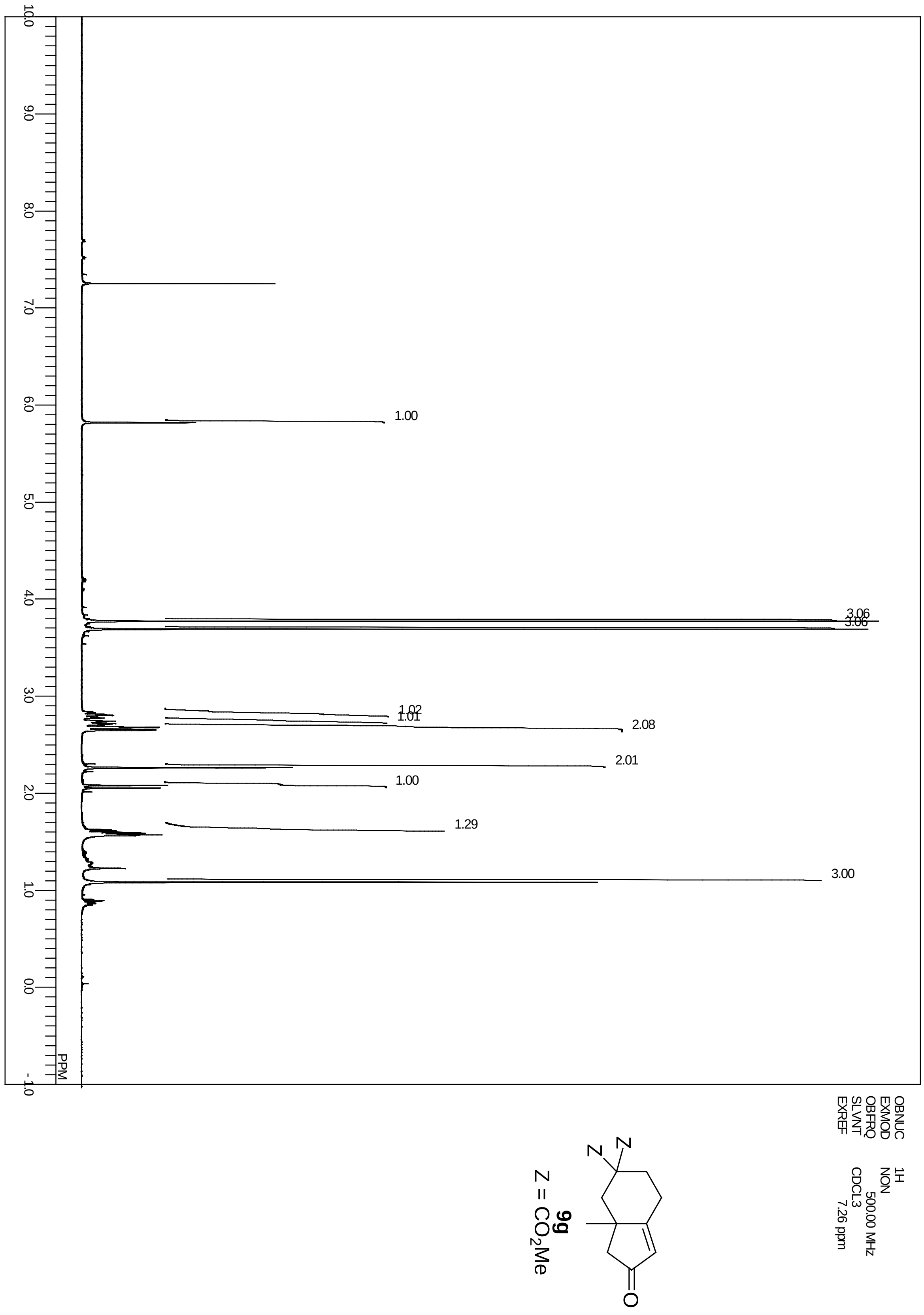
OBNUC 1H
EXMOD NON
OBFRQ 500.00 MHz
SLVNT CDCL3
EXREF 7.26 ppm
PPM
1.00
1.02
1.01
2.08
2.01
1.00
1.29
3.00
3.06
3.06
9g
Z = CO2Me

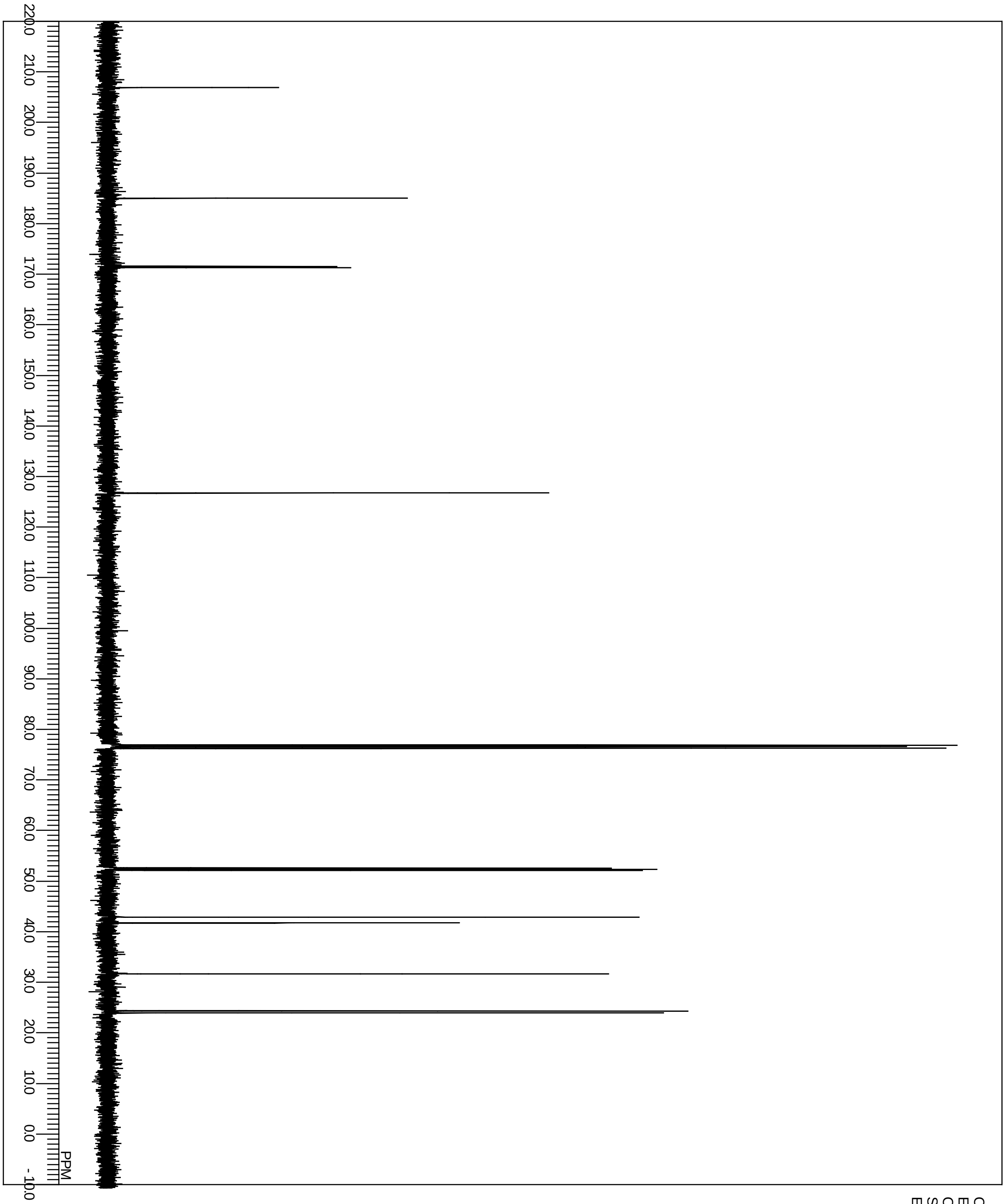
220.0 210.0 200.0 190.0 180.0 170.0 160.0 150.0 140.0 130.0 120.0 110.0 100.0 90.0 80.0 70.0 60.0 50.0 40.0 30.0 20.0 10.0 0.0 - 10.0
PPM
OBNUC 13C
EXMOD single_pulse_dec
OBFRQ 100.53 MHz
SLVNT CDCL3
EXREF 77.00 ppm

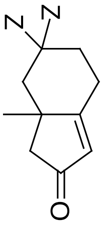
Z
Z
O

9g

Z = CO_2Me

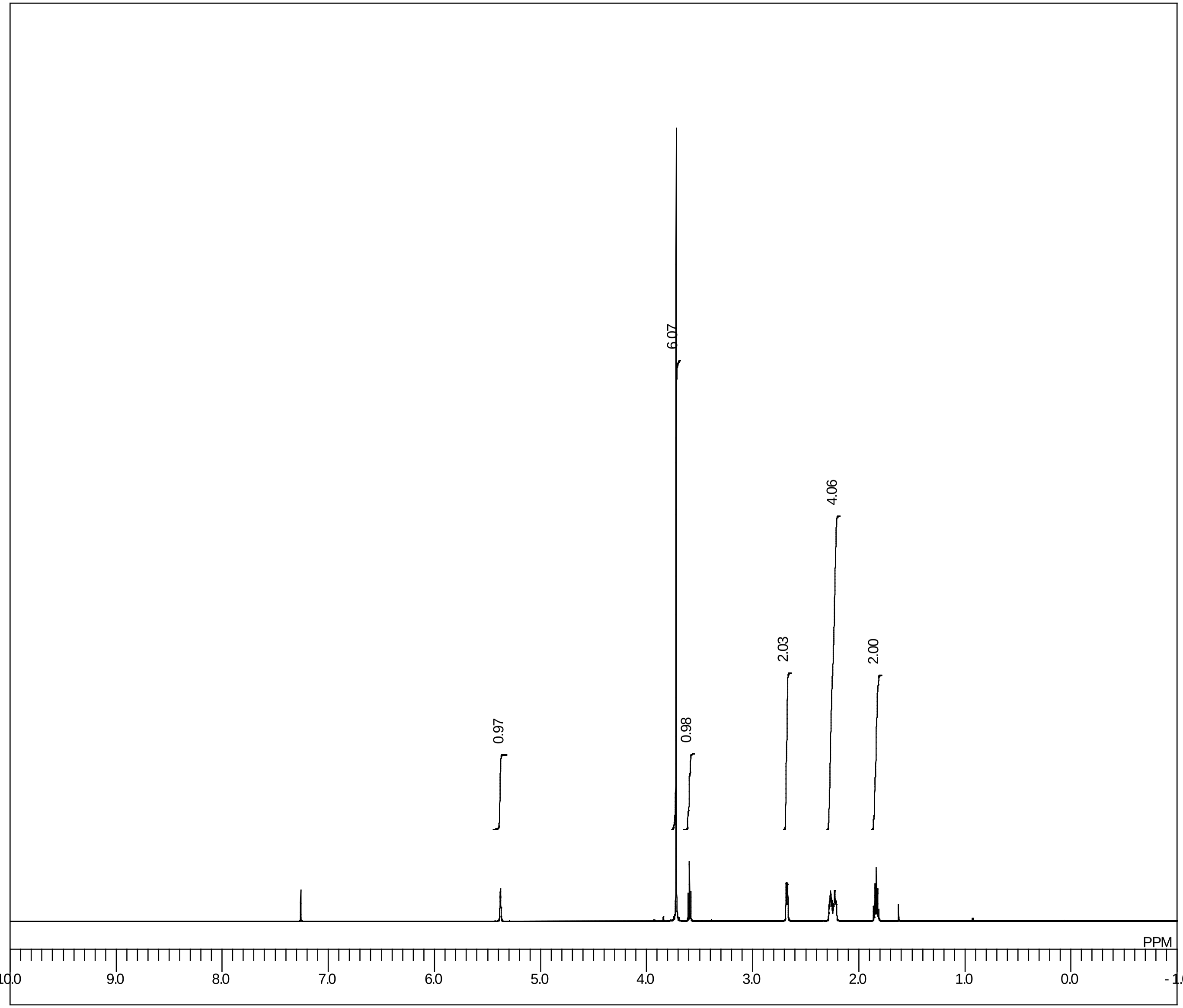
6.07
0.97
0.98
2.03
4.06
2.00
PPM
10.0
9.0
8.0
7.0
6.0
5.0
4.0
3.0
2.0
1.0
0.0
- 1.0
OBNUC 1H
EXMOD single_pulse.ex2
OBFRQ 600.17 MHz
SLVNT CDCL3
EXREF 7.26 ppm

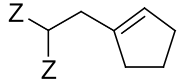
Z
Z

24

Z = CO_2Me

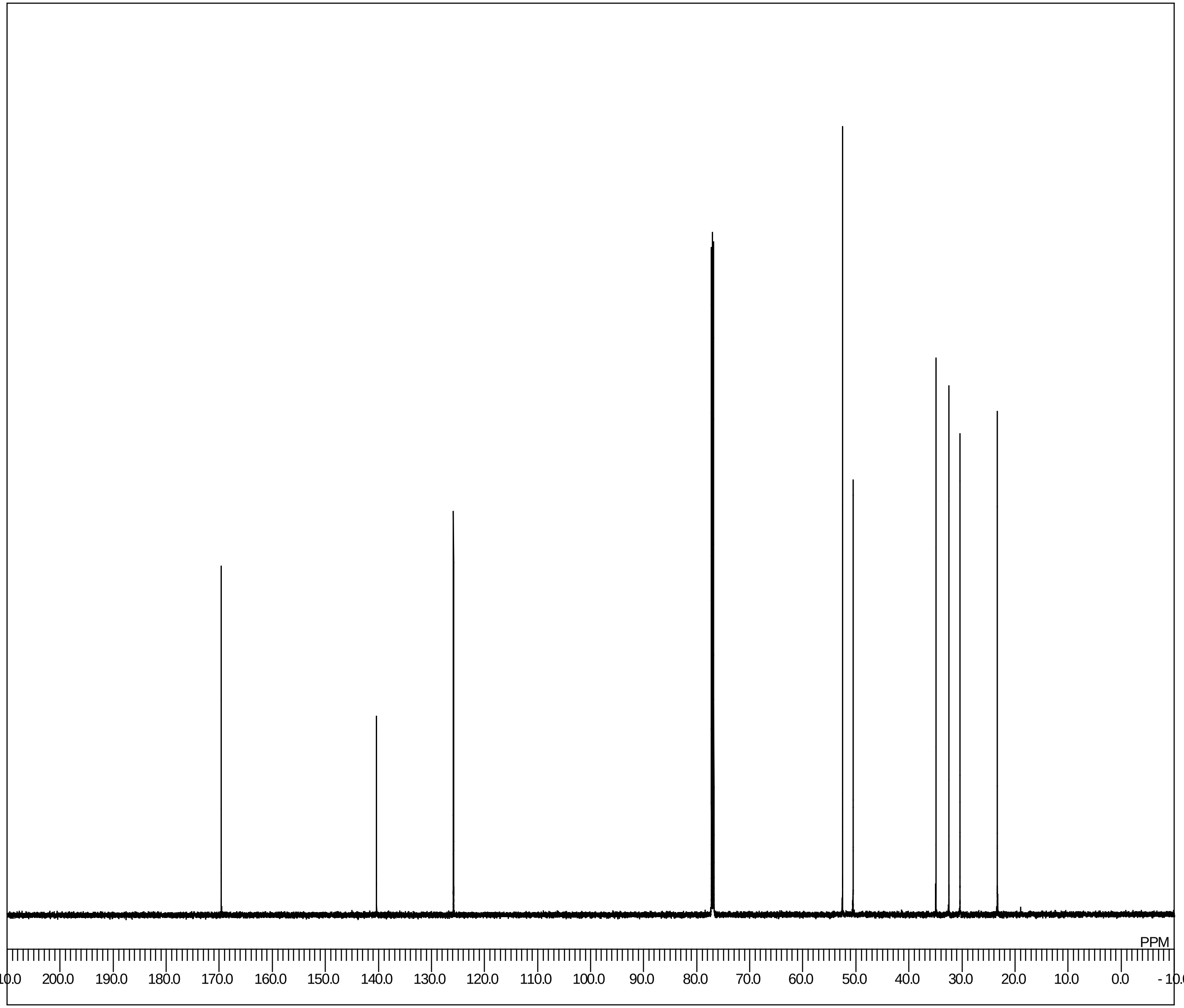

OBNUC 13C
EXMOD single_pulse_dec
OBFRQ 150.92 MHz
SLVNT CDCL3
EXREF 77.00 ppm

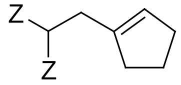

24
Z = CO_2Me

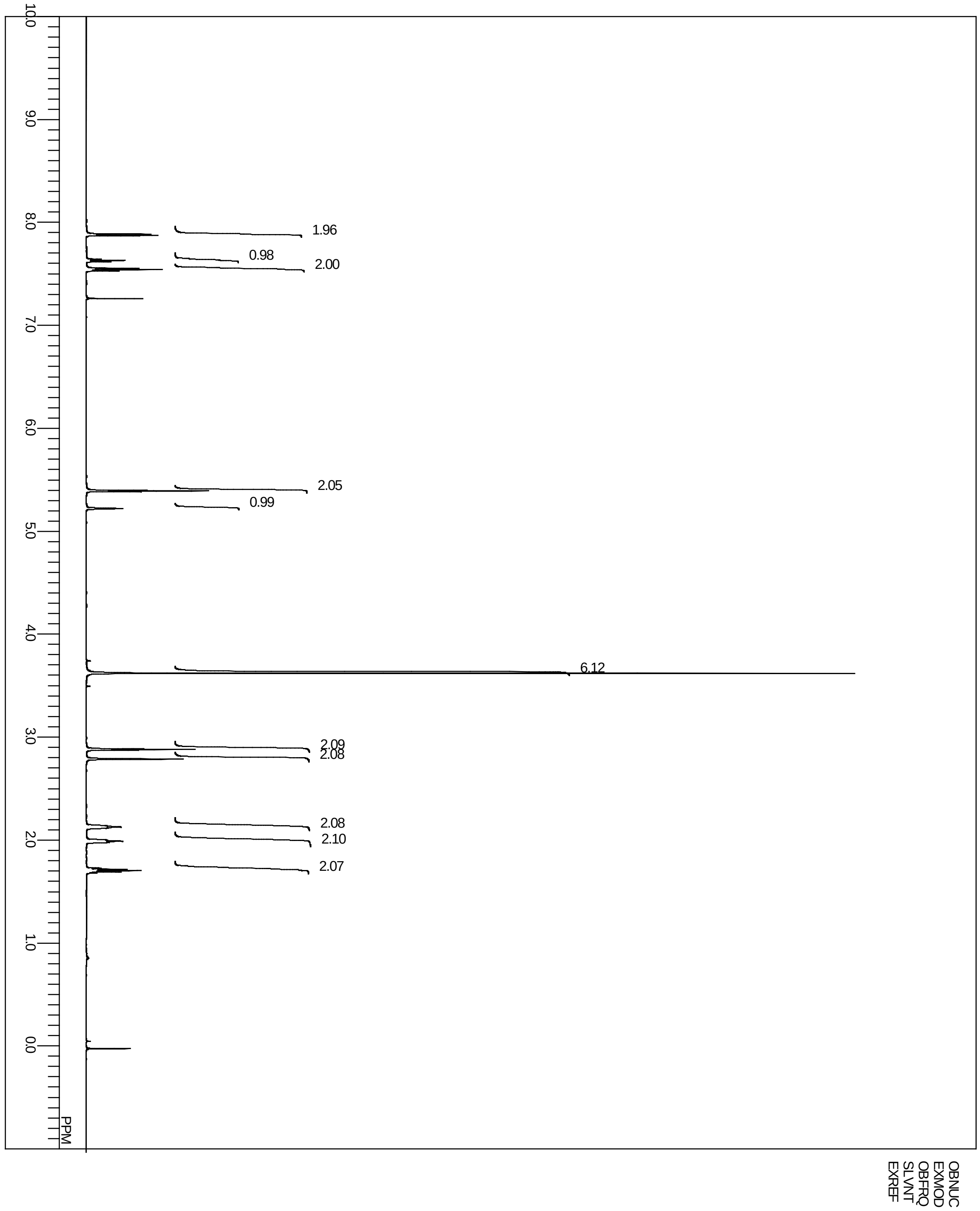
PPM
OBNUC 1H
EXMOD single_pulse.ex2
OBFRQ 600.17 MHz
SLVNT CDCL3
EXREF 0.00 ppm

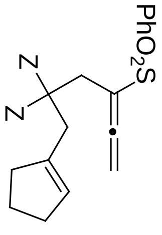
PhO2S
Z
Z

10

Z = CO_2Me

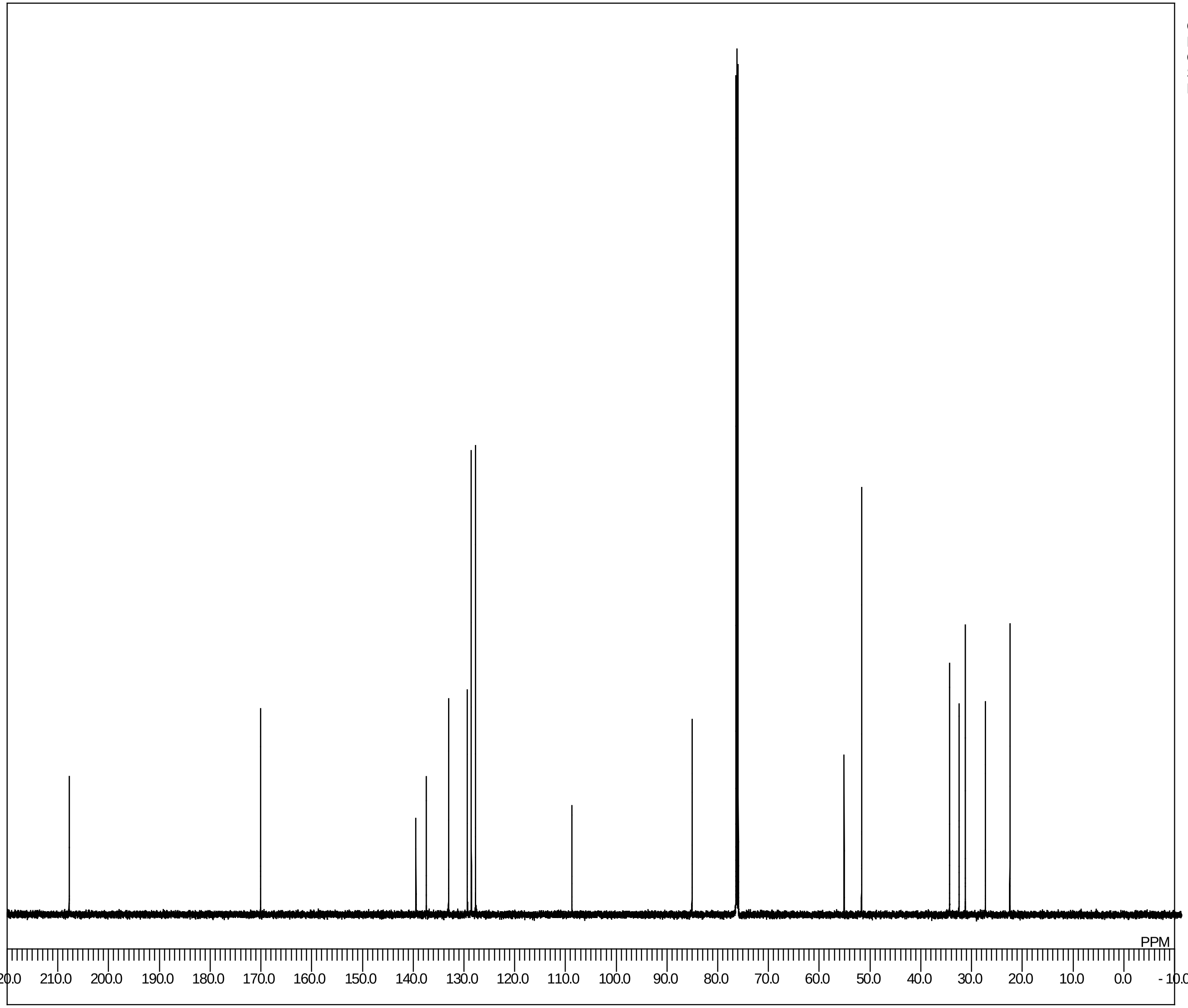

OBNUC 13C
EXMOD single_pulse_dec
OBFRQ 150.92 MHz
SLVNT CDCL3
EXREF 77.00 ppm

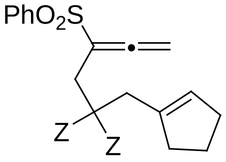

10
Z = CO_2Me

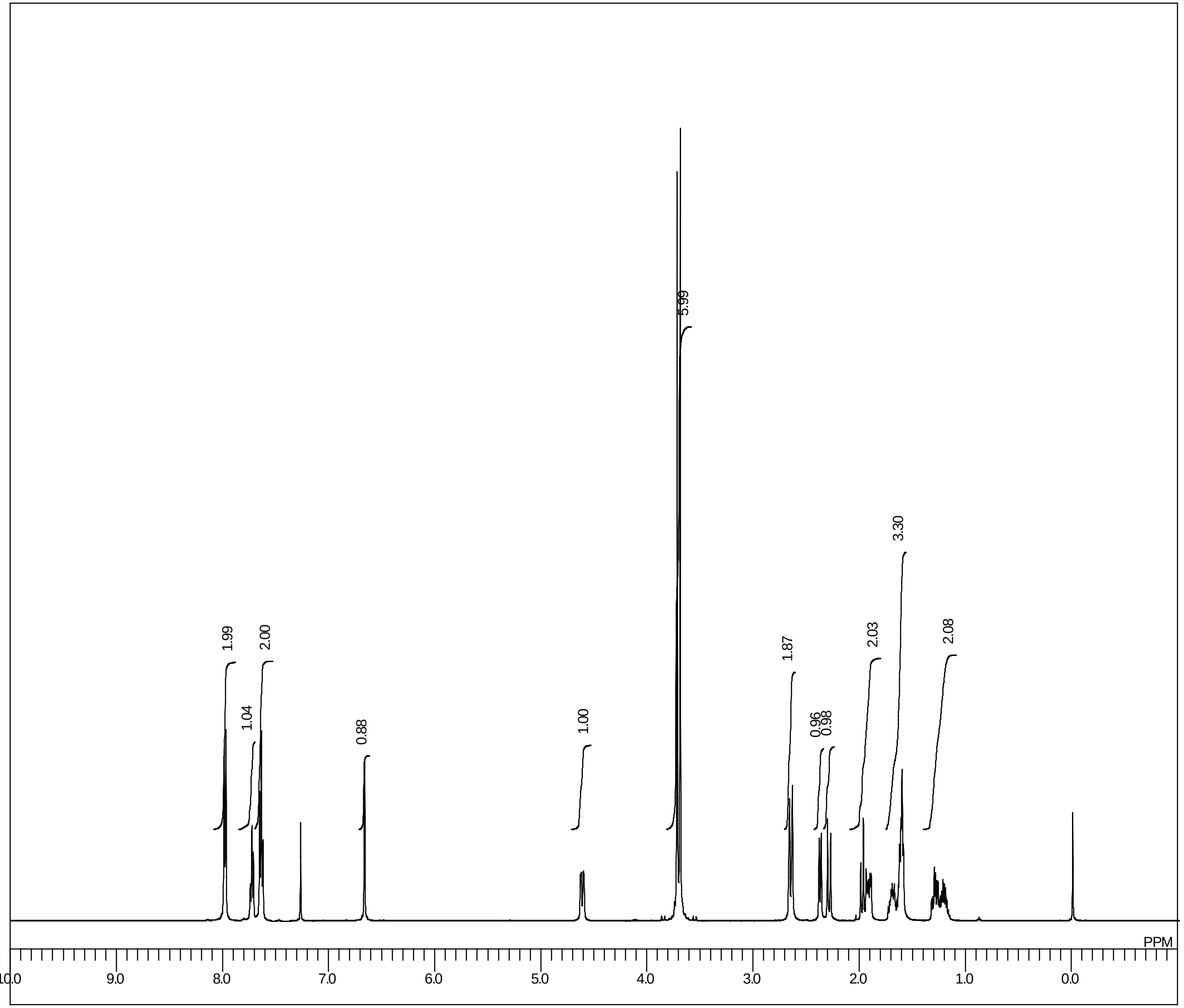

OBNUC	1H
EXMOD	NON
OBFRQ	500.00 MHz
SLVNT	CDCL3
EXREF	0.00 ppm

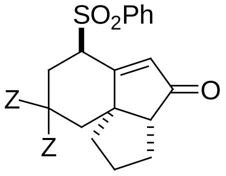

11
$Z = CO_2Me$

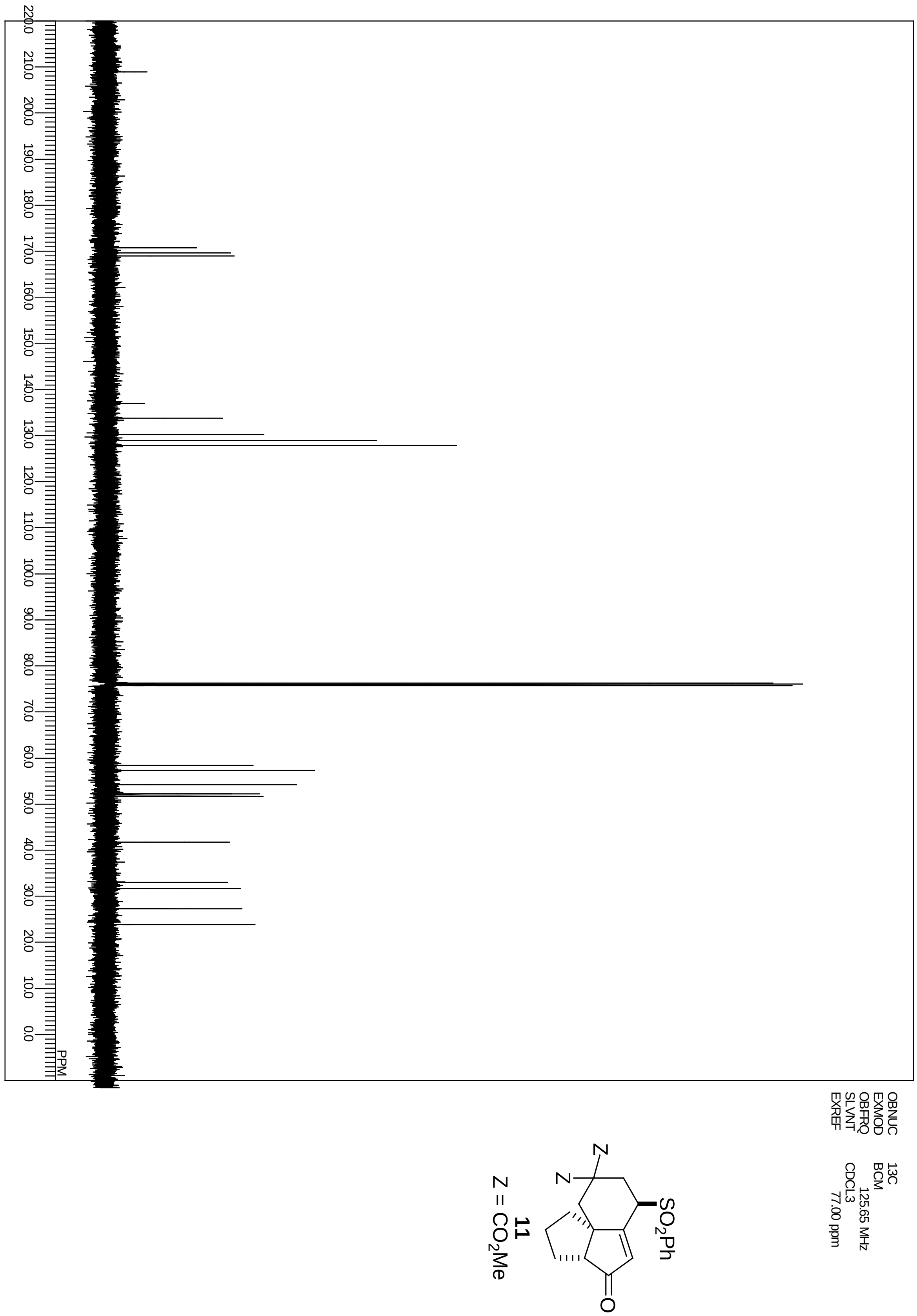
OBNUC 13C
EXMOD BCM
OBFRQ 125.65 MHz
SLVNT CDCL3
EXREF 77.00 ppm
PPM
220.0 210.0 200.0 190.0 180.0 170.0 160.0 150.0 140.0 130.0 120.0 110.0 100.0 90.0 80.0 70.0 60.0 50.0 40.0 30.0 20.0 10.0 0.0
SO2Ph
Z
Z
O
11
Z = CO2Me

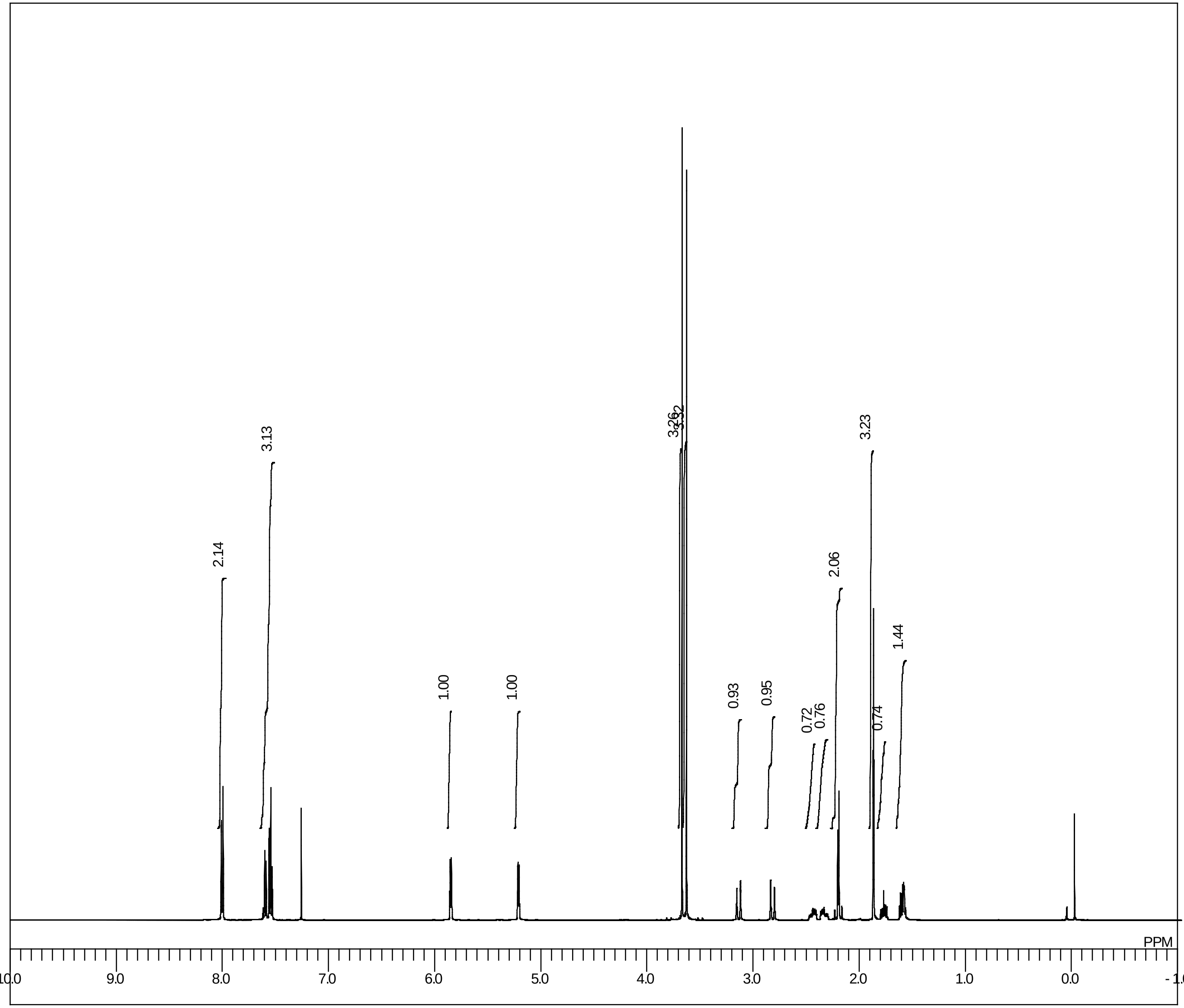

OBNUC	1H
EXMOD	NON
OBFRQ	500.00 MHz
SLVNT	CDCL3
EXREF	0.00 ppm

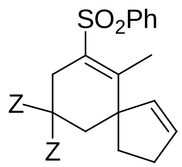

12

Z = CO_2Me

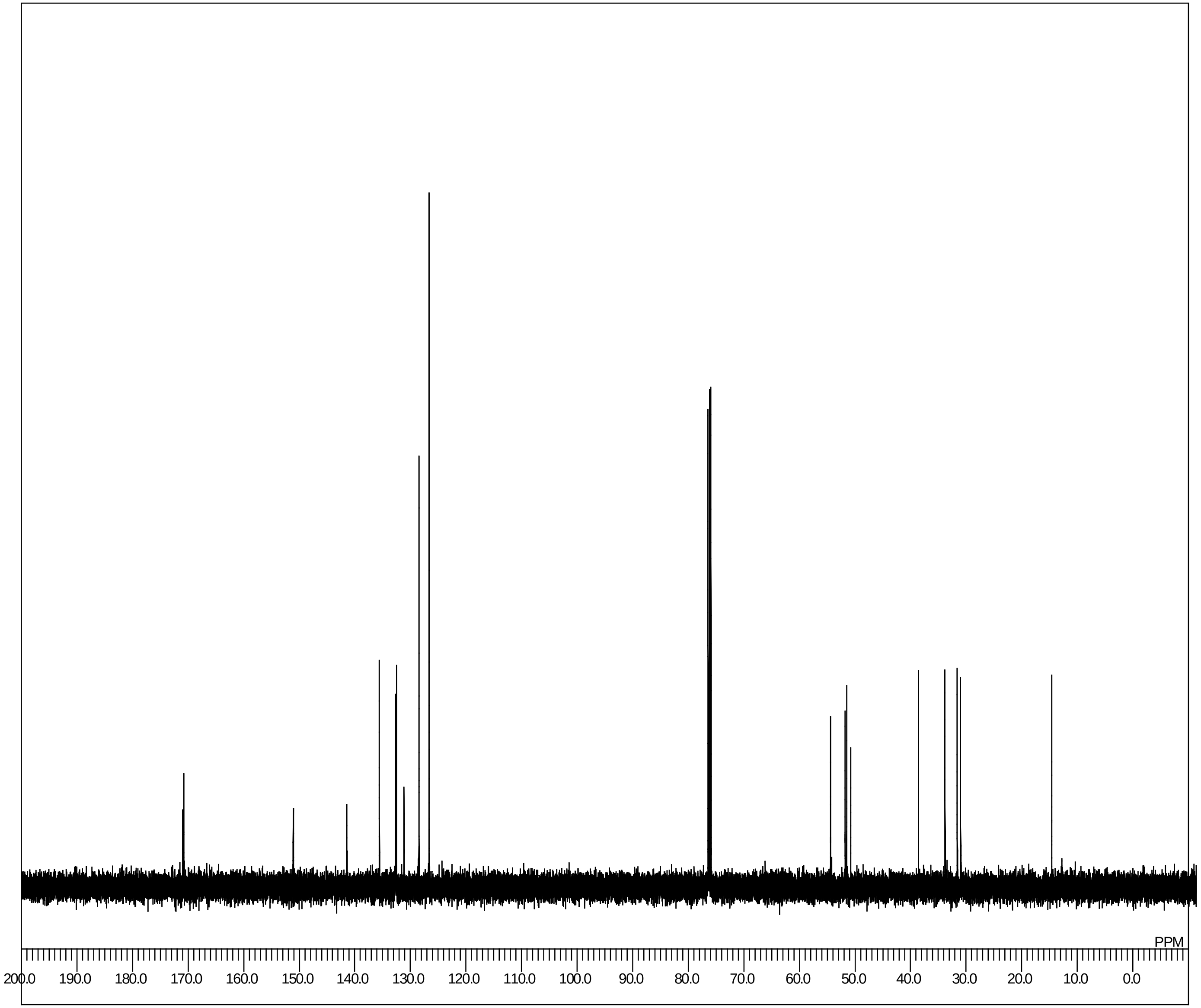

OBNUC 13C
EXMOD BCM
OBFRQ 125.65 MHz
SLVNT CDCL3
EXREF 77.00 ppm

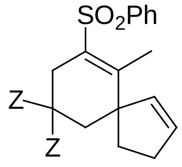

12
Z = CO_2Me
